# βA3/A1-crystallin regulates apical polarity and EGFR endocytosis in retinal pigmented epithelial cells

**DOI:** 10.1038/s42003-021-02386-6

**Published:** 2021-07-08

**Authors:** Peng Shang, Nadezda Stepicheva, Kenneth Teel, Austin McCauley, Christopher Scott Fitting, Stacey Hose, Rhonda Grebe, Meysam Yazdankhah, Sayan Ghosh, Haitao Liu, Anastasia Strizhakova, Joseph Weiss, Imran A. Bhutto, Gerard A. Lutty, Ashwath Jayagopal, Jiang Qian, José-Alain Sahel, J. Samuel Zigler, James T. Handa, Yuri Sergeev, Raju V. S. Rajala, Simon Watkins, Debasish Sinha

**Affiliations:** 1 Department of Ophthalmology, University of Pittsburgh School of Medicine, Pittsburgh, PA USA; 2 Dean McGee Eye Institute, University of Oklahoma Health Science Center, Oklahoma City, OK USA; 3 Wilmer Eye Institute, The Johns Hopkins University School of Medicine, Baltimore, MD USA; 4 Kodiak Sciences, Palo Alto, CA USA; 5 Institut de la Vision, INSERM, CNRS, Sorbonne Université, Paris, France; 6 National Eye Institute, National Institutes of Health, Bethesda, MD USA; 7 Department of Cell Biology and Center for Biologic Imaging, University of Pittsburgh School of Medicine, Pittsburgh, PA USA

**Keywords:** Cellular imaging, Imaging, Cell polarity

## Abstract

The retinal pigmented epithelium (RPE) is a monolayer of multifunctional cells located at the back of the eye. High membrane turnover and polarization, including formation of actin-based apical microvilli, are essential for RPE function and retinal health. Herein, we demonstrate an important role for βA3/A1-crystallin in RPE. βA3/A1-crystallin deficiency leads to clathrin-mediated epidermal growth factor receptor (EGFR) endocytosis abnormalities and actin network disruption at the apical side that result in RPE polarity disruption and degeneration. We found that βA3/A1-crystallin binds to phosphatidylinositol transfer protein (PITPβ) and that βA3/A1-crystallin deficiency diminishes phosphatidylinositol 4,5-biphosphate (PI(4,5)P_2_), thus probably decreasing ezrin phosphorylation, EGFR activation, internalization, and degradation. We propose that βA3/A1-crystallin acquired its RPE function before evolving as a structural element in the lens, and that in the RPE, it modulates the PI(4,5)P_2_ pool through PITPβ/PLC signaling axis, coordinates EGFR activation, regulates ezrin phosphorylation and ultimately the cell polarity.

## Abstract (web summary)

Shang et al. utilize a conditional *Cryba1* knockout mouse model to demonstrate a key role for βA3/A1-crystallin in retinal pigmented epithelium. As a result of βA3/A1-crystallin deficiency, the authors report EGFR endocytosis defects and actin network disruption which leads to polarity disruption and degeneration through PITP β/PLC signaling.

## Introduction

The retinal pigment epithelium (RPE) is a monolayer of highly polarized, differentiated, and mitotically quiescent cells, situated between the photoreceptors and the choriocapillaris in the retina, with diverse functions in retinal health, including roles in engulfment and digestion of shed photoreceptor outer segments, the visual cycle, and nutrient transport^1^. The function of RPE cells critically relies on their intensive membrane recycling activities, including phagocytosis, autophagy, and endocytosis^2^. In addition to their highly dynamic membrane activities, RPE cells have a highly polarized phenotype^3^. Like many other epithelial cell types, they have F-actin-based microvilli on their apical surface that serve as the platform for endocytosis, signal transduction, nutrient transport, etc^4^. With age and disease, the RPE microvilli become disorganized and atrophic^5^. The apical actin cytoskeletal architecture is organized by the membrane-cytoskeletal linker ezrin and its scaffolding protein-EBP50 (ezrin/radixin/moesin-binding phosphoprotein 50)^6^.

The RPE requires phosphoinositides (also known as phosphatidylinositol phosphates or PIPs), a group of phospholipids (PLs) that have fundamental roles in membrane dynamics and cytoskeleton organization, for cellular signaling^7^. In fact, dysregulation of PIP metabolism in photoreceptors has been reported to result in blindness^8–12^. While a few studies have focused on phosphatidylinositol-3-phosphate in membrane recycling in RPE cells^13^, the specific roles of different PIPs in RPE function and physiology remain uncertain^14,15^. For example, the specific role in the RPE of phosphatidylinositol-4,5-phosphate (PI(4,5)P_2_), the most abundant PIP and regulates multiple intracellular activities, including membrane remodeling and trafficking as well as cytoskeleton assembly^16^, is unknown. Importantly, PI(4,5)P_2_ has been shown to be critical for autophagy and phagocytosis, which are essential processes performed by the RPE for vision^16^.

Moonlighting proteins are multifunctional proteins that perform different functions in different cells or cellular locations^17^. The first described moonlighting proteins were crystallins, which provide transparency and refractivity of the lens^18^. In other tissues, αB-crystallin serves as a small heat shock protein^19^, while duck ε-crystallin is a lactate dehydrogenase^20^. Recently, we found that βA3/A1-crystallin is a moonlighting protein because in addition to its structural function in the lens, it is also expressed in retinal astrocytes, ganglion cells, and RPE cells^21,22^. In the RPE, βA3/A1-crystallin is required for normal lysosomal function, including maintenance of lysosomal pH, intracellular amino acid levels, and regulation of phagocytosis and autophagy^23–25^. We have shown that *Cryba1* cKO mice (conditional knockout of *Cryba1* specifically in the RPE) exhibit an age-related macular degeneration (AMD)-like phenotype^23,24,26,27^.

We have also found that βA3/A1-crystallin may play an important role in maintaining RPE polarity. This protein is enriched at the apical region of polarized RPE cells and is not expressed in non-polarized RPE cells, such as non-polarized cultured RPE cell lines^27^. Furthermore, RPE cells lacking *Cryba1*, the gene encoding βA3/A1-crystallin, undergo an epithelial-mesenchymal transition (EMT)-like process^27^. Importantly, βA3/A1-crystallin is unique among all crystallins in that two closely related polypeptides, βA3 and βA1, are produced from two different translation start sites on a single *Cryba1* mRNA by leaky ribosomal scanning^28^. The production of two polypeptides may contribute to the complexity of the intriguingly diverse functions of this moonlighting protein. By utilizing our well-characterized *Cryba1* cKO mouse model and RPE/choroid/sclera flatmounts, we herein suggest another important function of βA3/A1-crystallin: modulating the PI(4,5)P_2_ pool in RPE cells via the PITPβ/PLC signaling axis, with subsequent effects on cell polarity and EGFR signaling.

## Results

### *Cryba1* cKO RPE show age-related microvilli defects

**Fig. 1:**
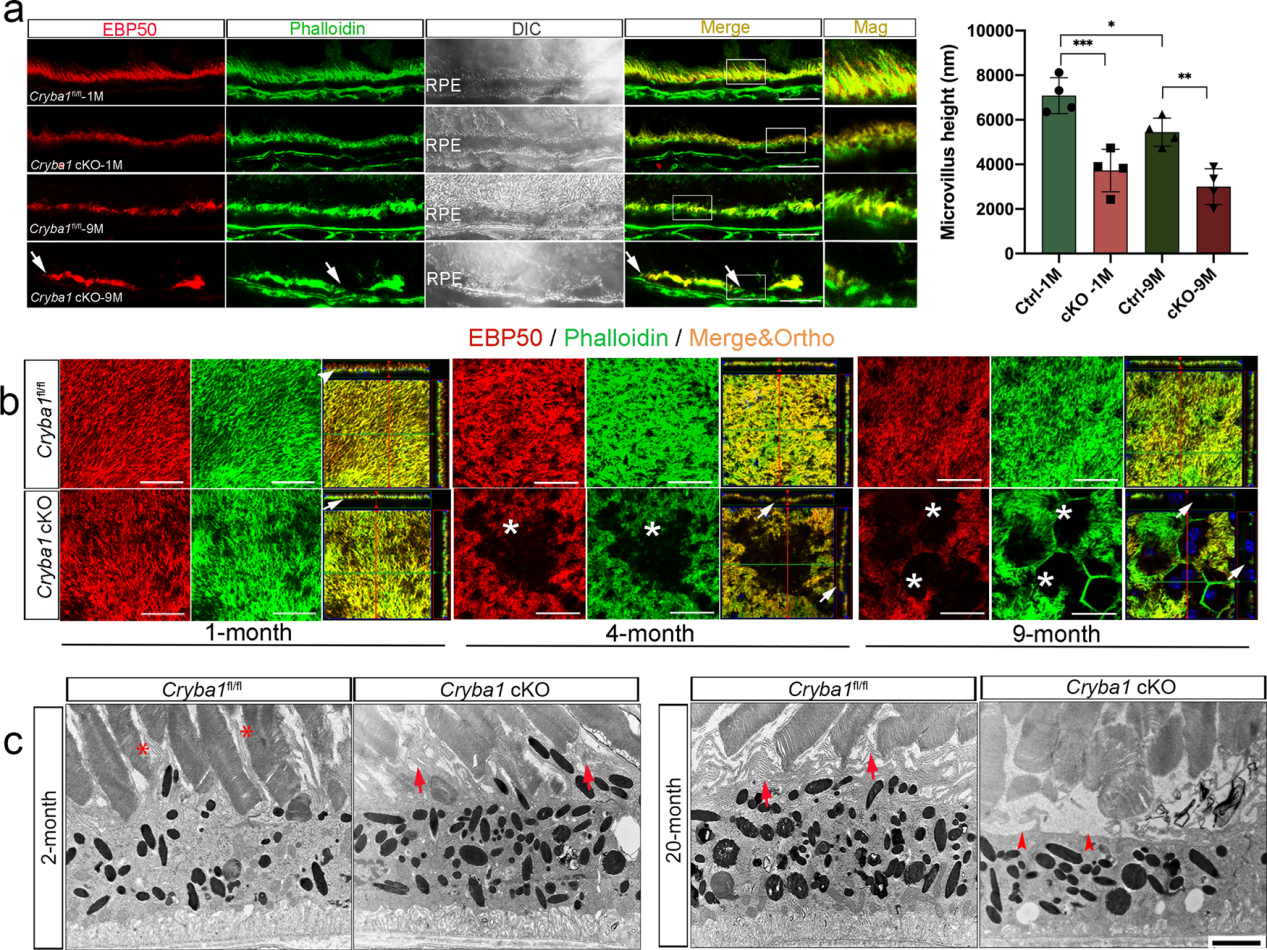
*Cryba1* cKO RPE show age-related microvilli defects. **a** Immunostaining for EBP50 (red) and F-actin (phalloidin, green) on retina sections showed disorganized microvilli in 1-month-old cKO RPE cells, and microvilli loss (arrows) in RPE cells of 9-month-old cKO mice compared to age-matched control. Mag: magnified area outlined in merged image. DAPI (blue). Scale bar: 20 μm. Graph shows the average microvilli height in control and cKO retina sections measured by the length measurement tool in ZEN software based on the staining results. For each biological repeat, three representative values from different RPE locations (center, middle, peripheral) were averaged as the microvillus height. Differential interference contrast (DIC) microscopy showed the area of each sample for imaging. **b** Z-stack imaging for EBP50 (red) and F-actin (phalloidin, green) with an orthogonal projection showing upright and abundant microvilli in 1-month-old control (arrowhead), but not in cKO RPE cells (arrow). In 4-month-old mice, some cKO RPE cells presented collapsed microvilli indicated by sinking F-actin and EBP50 in the *z*-direction (arrows). Some RPE cells of 9-month-old cKO mice (asterisks) lacked microvilli completely. Scale bar: 20 μm. **c** TEM images of cKO and control retina sections. Two-month-old control mice exhibited microvilli very well interdigitated with photoreceptor outer segments (asterisks). Arrows show disorganized microvilli of RPE cells in 2-month-old cKO and 20-month-old control mice. Arrowheads show microvilli loss in some RPE cells in 20-month-old cKO mice. Scale bar: 4 μm. Statistical analysis was performed using one-way ANOVA. *P < 0.05, **P < 0.01, ***P < 0.001, *n* = 4.

We investigated the RPE microvilli on retinal sections and RPE flatmounts from *Cryba1* cKO and age-matched floxed control mice by immunostaining for F-actin and EBP50. At 1 month of age, the apical microvilli of RPE cells in floxed mice are robust and well interdigitated with photoreceptor outer segments, while in *Cryba1* cKO mice they are disorganized. This disorganization became more severe with age (Fig. 1a, b). By 4 months of age, microvilli in RPE cells from *Cryba1* cKO mice had collapsed, progressing to near complete loss of microvilli in some RPE cells by 9 months. These abnormalities can be visualized by the orthogonal projection of z-stack confocal images on RPE flatmounts where the XY panels are taken from the same apical plane (Fig. 1b). The lower magnification images of retina cryosections and RPE flatmounts showed a patchy pattern of microvilli abnormalities in cKO RPE cells (Supplementary Fig. 1a, b). Ultrastructurally, the RPE exhibited disorganized interdigitation of microvilli with photoreceptor outer segments in 2-month-old *Cryba1* cKO retinas, and complete loss of microvilli in some RPE cells by 20 months (Fig. 1c). It is worth noting that the control RPE cells also showed mild age-related microvilli disorganization (Fig. 1a–c). Our data are in agreement with another group that showed progressive microvilli atrophy (shortening) in aging rats^5^.

**Fig. 2:**
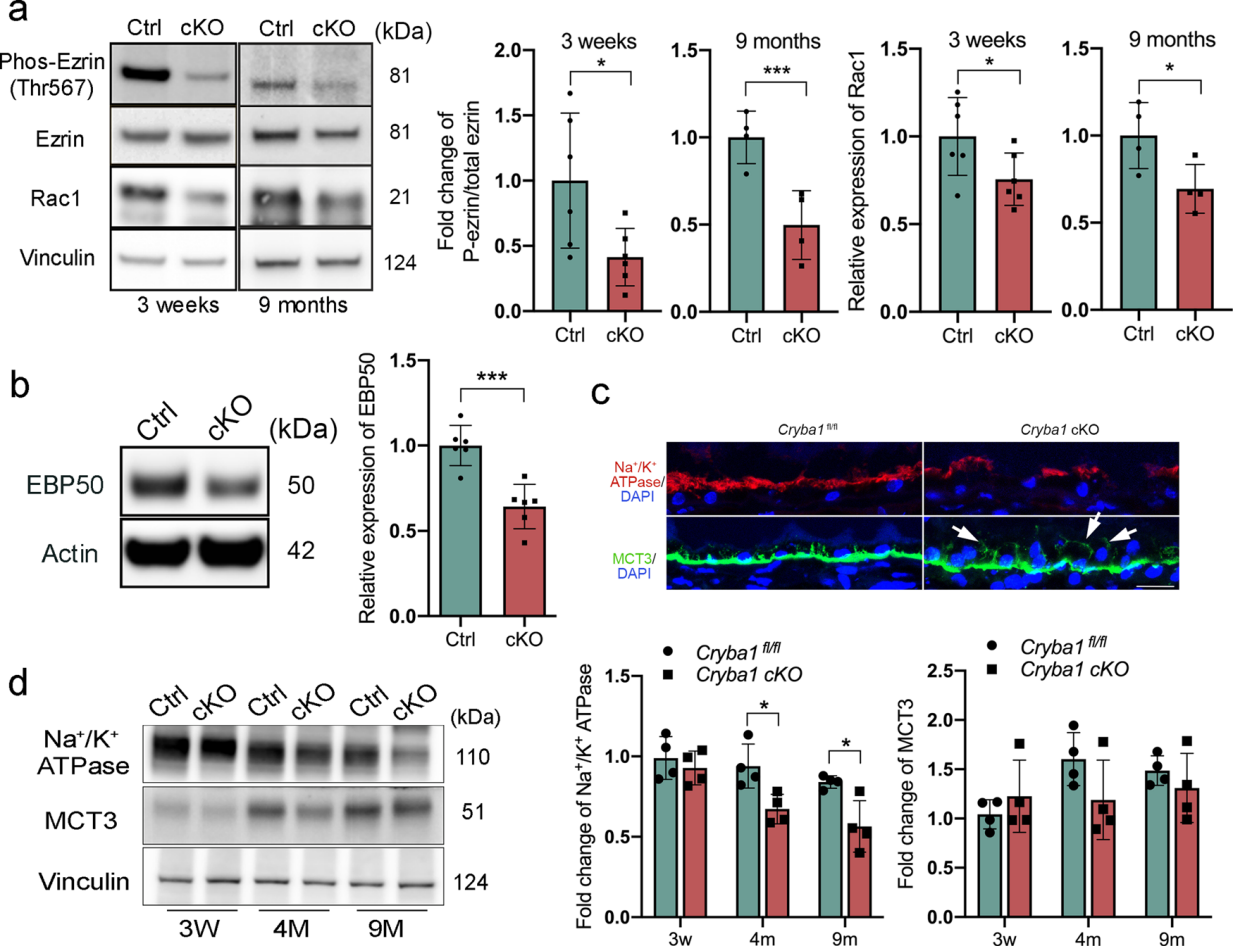
Disrupted apical-basal polarity in RPE cells lacking *Cryba1*. **a** Western blot analysis of 3-week and 9-month-old RPE extracts showing that ezrin phosphorylation and Rac1 expression were reduced in cKO RPE cells at both ages. **b** Western blot analysis of 4-month-old RPE extracts confirmed downregulation of EBP50 protein in cKO RPE cells. **c** Immunostaining for Na^+^/K^+^ ATPase and MCT3 on sections of 9-month-old retina showed reduced Na^+^/K^+^ ATPase and mis-located MCT3 in *Cryba1* cKO RPE cells (indicated by arrows). Scale bar: 20 μm. **d** Western blot analysis of RPE extracts from control and cKO mice at different ages showed reduced Na^+^/K^+^ ATPase; however, there was no significant change in MCT3 abundance in cKO RPE. Statistical analysis was performed using either a two-tailed unpaired Student’s *t*-test (**a**, **b**) or two-way ANOVA (**d**). *P < 0.05, ***P < 0.001, *n* = 4~6.

To investigate the mechanism of microvilli atrophy, we checked the levels of ezrin phosphorylation since its scaffold protein, EBP50, only binds to phosphorylated ezrin to bring microfilaments to the plasma membrane^29^. We found that ezrin phosphorylation (Thr567) was substantially decreased in 3-week-old *Cryba1* cKO RPE cells (Fig. 2a). The small GTPase Rac1, which has been shown to be activated by ezrin that has been phosphorylated at Thr567^30,31^, was also reduced in the RPE from cKO mice. Both changes persisted through at least 9 months of age (Fig. 2a). These results indicate that lack of βA3/A1-crystallin impairs microvilli organization through a decrease in ezrin phosphorylation. EBP50 protein was also decreased in the RPE from *Cryba1* cKO mice compared to controls (Fig. 2b). Furthermore, we detected a significant reduction in Na^+^/K^+^-ATPase, an apical marker for RPE cells^32^, at 4 and 9 months old, but not at 3 weeks of age in RPE cells from the mutant mice (Fig. 2c, d). The levels of monocarboxylate transporter 3 (MCT3), which is normally localized to the RPE basolateral membrane^33^, were not significantly different in *Cryba1* cKO RPE cells compared to controls. However, in the 9-month-old cKO cells, MCT3 lost its basolateral localization and was present in the cytoplasm and at the apical domain, indicating that RPE polarity was disrupted in RPE cells lacking βA3/A1-crystallin (Fig. 2c, d).

**Fig. 3:**
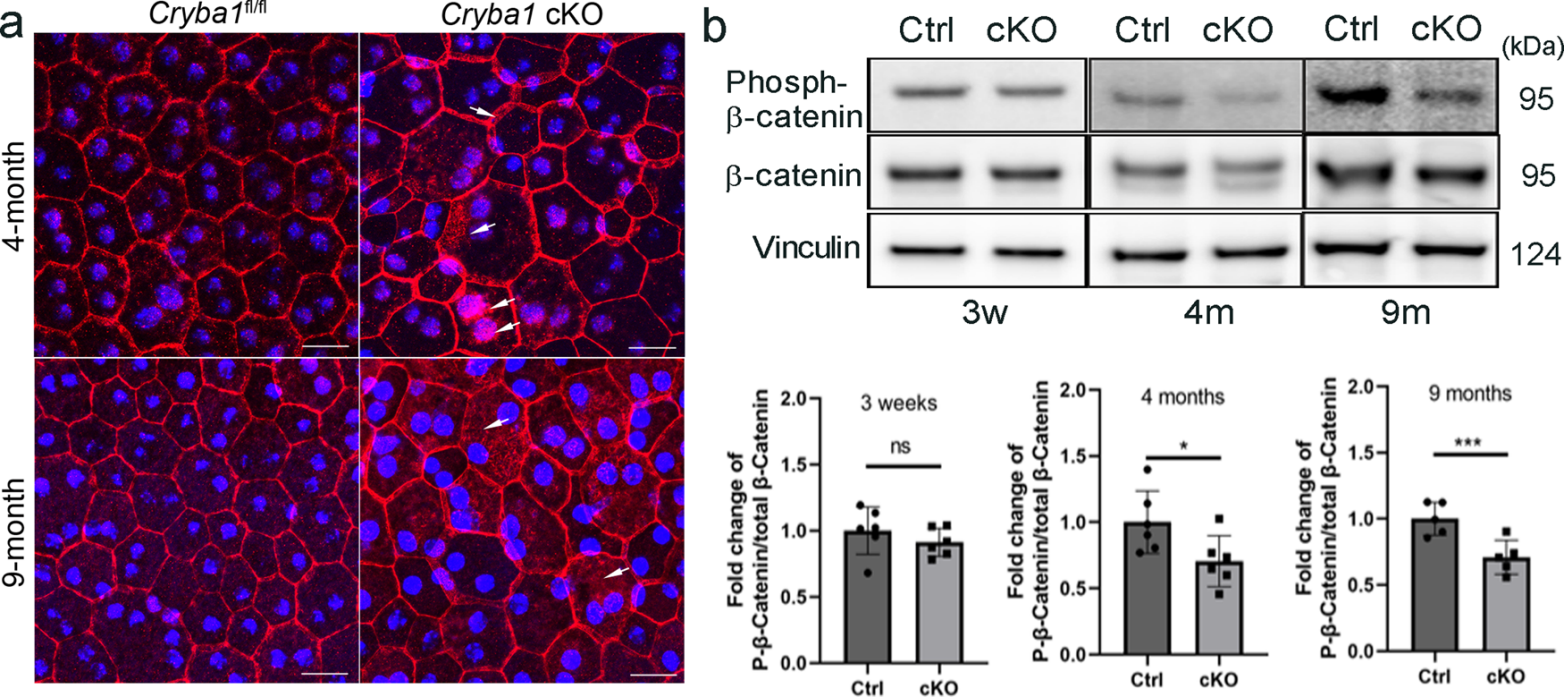
Activated β-catenin in *Cryba1* cKO RPE. **a** RPE flatmounts prepared from 4-month and 9-month-old *Cryba1* cKO and *Cryba1*^fl/fl^ control mice were stained with anti-β-catenin antibody (red) and DAPI (blue). Arrows show cytoplasmic and nuclear β-catenin in cKO RPE cells. Scale bar: 20 μm. **b** RPE lysates of *Cryba1* cKO and *Cryba1*^fl/fl^ control mice at different ages were collected and analyzed by western blotting for phosphorylated β-catenin and total β-catenin. Vinculin was used as an internal control. Graphs show decreased phosphorylated β-catenin at 4 and 9 months of age in the cKO cells. Statistical analysis was performed using a two-tailed unpaired Student’s *t*-test. *P < 0.05, ***P < 0.001, *n* = 5~6.

Other important factors that are required to maintain RPE polarity are adherens and tight junctions^34,35^. β-catenin, an adherens junction component that interacts with cadherins or actin in normal conditions, is anchored to cell membranes^36^. Studies have shown that β-catenin plays a role in cell–cell contact and the EMT process in native mouse RPE^27,37^. In RPE flatmounts, β-catenin immunostaining showed partial translocation of β-catenin to the cytoplasm and nucleus in RPE cells of both 4- and 9-month-old *Cryba1* cKO mice, while it remained restricted to the plasma membrane in RPE cells from age-matched control mice (Fig. 3a). We also observed that β-catenin phosphorylation was significantly decreased in the RPE cells of *Cryba1* cKO mice compared to age-matched control RPE cells, starting at 4 months of age (Fig. 3b). Phosphorylated β-catenin usually recruits ubiquitin E3 and is subsequently degraded by the proteasome. Non-phosphorylated β-catenin is able to translocate to the nucleus and initiate transcription of EMT-related genes^36^. It can be speculated that in *Cryba1* cKO RPE cells, the decreased β-catenin phosphorylation allows more β-catenin translocation to the cytoplasm and nucleus, thereby initiating the EMT-like processes. We previously showed that RPE cells from *Cryba1* cKO mice exhibit elevated mRNA for the EMT transcription factors Snai1 and Snai2, elevated vimentin protein, and loss of E-cadherin with dynamic reorganization of the actin cytoskeleton by 5 months of age^27^. Interestingly, the phosphorylation level of β-catenin was not different in the cKO RPE at 3 weeks of age (Fig. 3b), indicating that activated β-catenin signaling in cKO RPE cells is likely to be a secondary effect of the cytoskeletal disorganization.

### EGFR activation and internalization is compromised in *Cryba1* cKO RPE

**Fig. 4:**
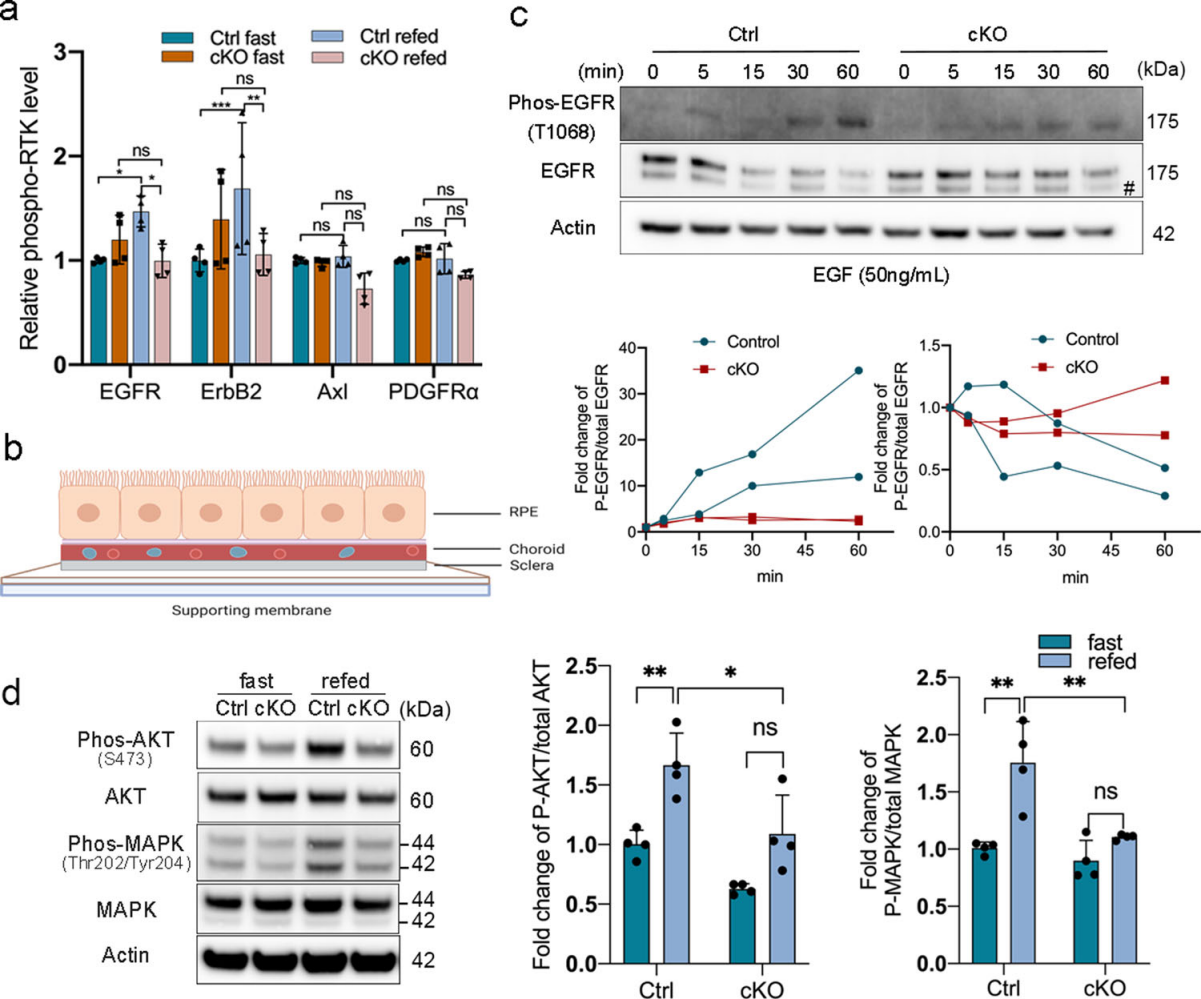
Compromised EGFR activation in *Cryba1* cKO RPE. **a** RPE lysates from 2-month-old control and cKO mice fasted or fasted and then refed were isolated for a phospho-RTK array assay. Phosphorylated EGFR and ErbB2 were decreased in refed cKO RPE cells compared to refed floxed controls. *n* = 4. **b** A diagram showing RPE–choroid–sclera flatmount preparation. **c** RPE flatmounts of 2-month-old control and cKO mice stimulated with EGF for times indicated were analyzed by western blot. cKO RPE cells presented less phosphorylated EGFR in response to EGF stimulation at each time point. While total EGFR gradually decreased after ligand stimulation in control RPE cells, EGFR was not reduced in cKO RPE cells. Graphs show the EGFR phosphorylation level and total EGFR level in both cell types. Immunoblots are representative of two independent experiments. #: non-specific band. **d** Phosphorylated AKT and MAPK levels in RPE from fasted or refed mice were measured by western blot. Both phosphorylated AKT and MAPK were significantly upregulated in control RPE after refeeding, but not in cKO RPE. *n* = 3. Statistical analysis was performed using two-way ANOVA (**a**, **d**). *P < 0.05, **P < 0.01, ***P < 0.001, ns: not significant.

Since phosphorylated ezrin also transduces extracellular signals by binding to receptor tyrosine kinases (RTKs) at the cell membrane^38,39^, and RTKs are important signaling transducers that initiate signaling cascades for various intracellular activities^40^, we investigated if loss of βA3/A1-crystallin affects RTK activation in the RPE. A phospho-RTK array was performed on RPE lysates from 2-month-old floxed control and *Cryba1* cKO mice (Supplementary Fig. 2a). In order to stimulate RTKs and reduce individual variation, all mice were fasted for 36 h with or without subsequent refeeding for 3 h prior to the assay. We found that the phosphorylation of epidermal growth factor receptor (EGFR or ErbB1) and ErbB2, two members of the ErbB family of RTKs, was lower in *Cryba1* cKO compared to floxed control RPE cells from mice that were refed for 3 h following fasting (Fig. 4a). However, other phosphorylated RTKs, such as platelet-derived growth factor subunit A (PDGFRα) (Fig. 4a), either showed no significant difference between the groups, or had low abundance in RPE cells (Supplementary Fig. 2a). ErbB2, which has no identified activating soluble ligand and usually forms a heterodimer with other ErbB members^41^, may form a heterodimer with EGFR in RPE cells. Thus, *Cryba1* deficiency in the RPE did not alter the phosphorylation of all RTKs, but rather, specifically regulated the phosphorylation of EGFR and its interactive RTK (ErbB2). These data are supported by our previous observation that βA3/A1-crystallin interacted with EGFR in a proteome high-throughput array (CDI NextGen Proteomics Laboratories, Inc.)^23^. While EGFR is a well-studied RTK in cancer cell biology^42^, little is known about its function in RPE cells. The specific effect of βA3/A1-crystallin on EGFR activation implies an important physiological role of βA3/A1-crystallin/EGFR signaling in RPE.

In order to confirm that EGFR phosphorylation in cKO RPE cells is defective, we established an in vitro model to culture RPE explants from mice (see Methods for details). This method maintains RPE cells in their original milieu, while allowing in vitro manipulations. The RPE–choroid–sclera (RCS) complexes were carefully flattened on a supporting membrane and put into culture (Fig. 4b). RPE cell viability was determined by the level of the cell death marker, receptor interacting protein kinase 1 (RIP1) (Supplementary Fig. 2b). *Cryba1* cKO RPE cells in situ had reduced levels of phosphorylated EGFR following EGF stimulation. While total EGFR levels were decreased after ligand stimulation in floxed control RPE cells due to degradation^43^, their levels were unchanged in *Cryba1* cKO RPE cells (Fig. 4c and Supplementary Fig. 2c) because of compromised EGFR activation. Interestingly, we observed that EGFR is activated at later time points (30–60 min post EGF stimulation) in cultured RCS complexes compared to the mouse RPE cell line (HPV E6/E7). The RCS model data are in sharp contrast to several previous reports using multiple cell lines^44,45^ including HPV E6/E7 RPE cells, that maximum EGFR activation occurs within 5–10 min after ligand stimulation (Supplementary Fig. 2d and Fig. 4c).

To better delineate if an EGFR activation defect would influence downstream signaling pathways, we measured the phosphorylation of AKT1 and MAPK, both well-known EGFR downstream effectors^46^, and found that their phosphorylation was upregulated in native RPE cells of control mice 3 h after refeeding, but not in *Cryba1* cKO RPE cells (Fig. 4d).

**Fig. 5:**
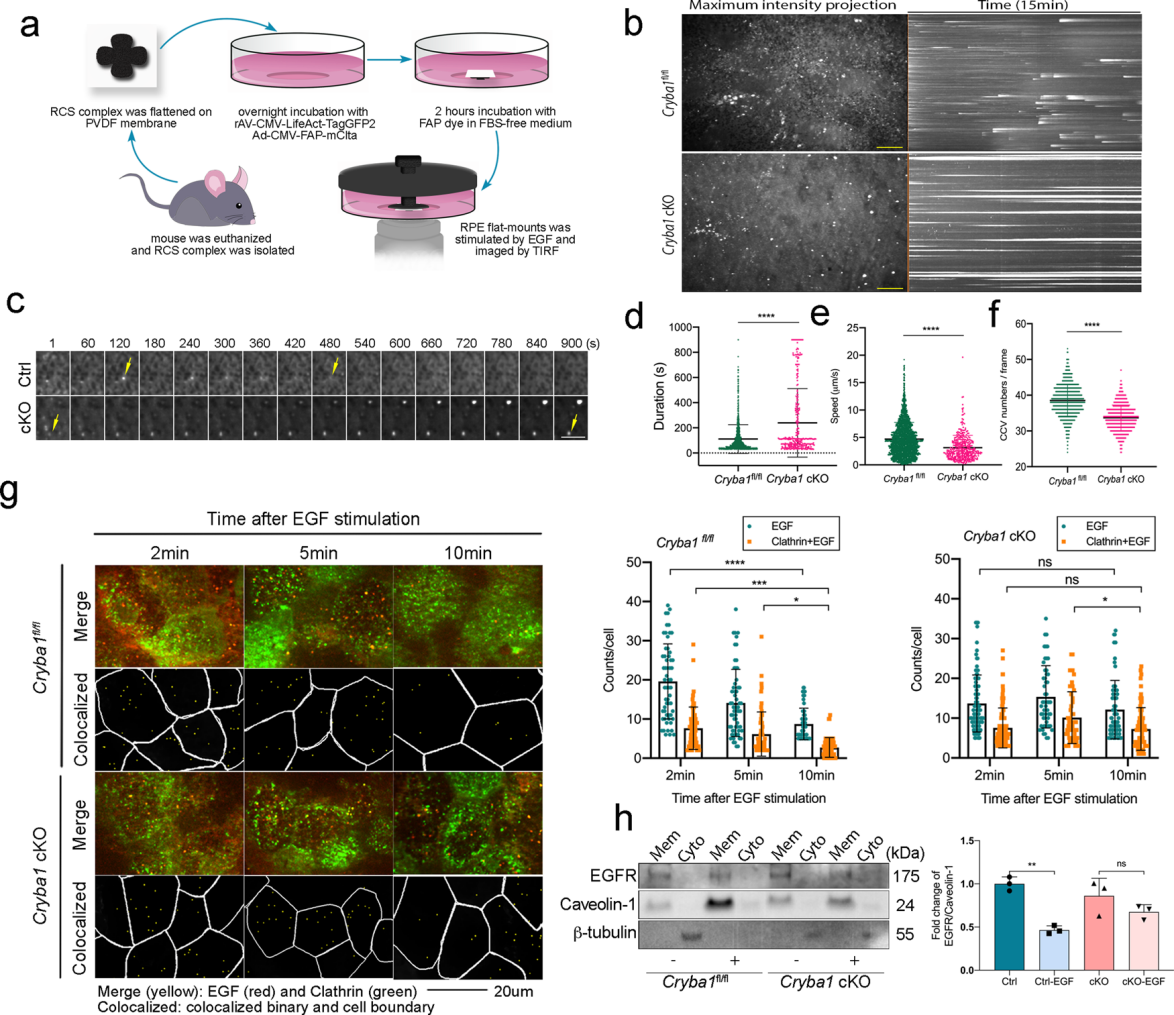
Compromised clathrin-mediated EGFR internalization in *Cryba1* cKO RPE. **a** A diagram showing sample preparation for TIRF and HiLo imaging of endocytosis on the apical side of RPE cells in situ. **b** RPE flatmounts from 2-month-old control and cKO mice were infected with Ad-CMV-m*Clta*-FAP and rAV-CMV-LifeAct-TagGFP2 and stimulated with EGF. Representative time-lapse TIRF imaging of clathrin from four biological repeats is shown in maximum intensity projection (*x*–*y* axis). The corresponding kymographs (*x*–*t* axis projection) show that a significant number of CCPs in *Cryba1* cKO RPE were stagnant at the apical side of the membrane and did not pinch off from the plasma membrane, indicated by long lines. Scale bar: 10 μm. **c** A representative CCP from each cell type was tracked over the acquisition time. As time proceeded, the CCP in the control RPE cell pinched off from the membrane after showing up in the imaging frame as the arrows show. However, the CCP in the cKO RPE cell was stuck at the cell membrane during the acquisition time window. Scale bar: 5 μm. **d** Duration of CCPs in cKO (CCP number = 310) and control RPE (CCP number = 1217) captured by TIRF. CCPs with a duration time less than 30 s were filtered out for this analysis. **e** Speed of each CCV in control (CCV number = 1639) and cKO (CCV number = 590) captured by HiLo microscopy were plotted. **f** CCVs were counted in each frame of live-cell imaging of *Cryba1* cKO RPE and control RPE samples. *n* (frames) = 1000. **g** RPE flatmounts from 2-month-old control and cKO mice infected with Ad-CMV-m*Clta*-FAP and rAV-CMV-LifeAct-TagGFP2 were collected at times indicated after Alexa-Fluo-555 EGF stimulation and imaged by TIRF microscopy. EGF and EGF-loaded CCPs represented by colocalization of clathrin and EGF were counted, respectively. *n* (cell number) = 54–90. Data suggest a failure of EGF-loaded CCPs to internalize into the cell in the cKO RPE. **h** Membrane and cytoplasmic protein fraction were separated from RPE flatmounts stimulated with 50 ng/mL EGF for 30 min. Western blot analysis were performed to detect EGFR level in membrane fraction from different cell types. Caveolin-1 and β-tubulin were used as membrane and cytoplasm marker, respectively. The plot shows the EGFR level in membrane fraction significantly decreased in control RPE cells due to internalization, but not in *Cryba1* cKO RPE cells. *n* = 3. Statistical analysis was performed using a two-tailed unpaired Student’s *t*-test (**d**–**f**), two-way ANOVA (**g**), and one-way ANOVA (**h**). *P < 0.05, **P < 0.01, ***P < 0.001, ****P < 0.0001, ns: not significant.

Since EGFR activation is perturbed in *Cryba1* cKO RPE cells, we asked if clathrin-mediated endocytosis of EGFR is also compromised. The high melanin content and autofluorescence of the RPE impairs visualization of the RPE apical surface^47^. Therefore, we developed a live-cell technique to image clathrin-mediated endocytosis from the apical side of RPE cells using total internal reflection fluorescence (TIRF) microscopy. As shown in Fig. 5a, RPE flatmounts were cultured in glass-bottom dishes and infected with two recombinant human serotype 5 adenovirus vectors with deleted E1/E3. One of the vectors contained a clathrin light chain A construct with a CMV promoter and a fluorogen-activated peptide (FAP) reporter (Ad-CMV-FAP-m*Clta*). This reporter only fluoresces after being activated by a fluorogen such as MG-ester, a far-red, cell-permeable FAP fluorogenic dye. The other vector contained a LifeAct-GFP fusion protein to fluorescently label the F-actin cytoskeleton. Infection efficiency was similar for control and *Cryba1* cKO RPE flatmounts (Supplementary Fig. 3a). After starving in fetal bovine serum (FBS)-free growth medium, RPE flatmounts were stimulated with EGF. To exclude the possibility that the clathrin signals were from non-specific autofluorescent particles, RPE flatmounts were incubated with or without MG-ester dye. Those without MG-ester dye showed no signal, which confirmed specificity (Supplementary Fig 3b). The TIRF time-lapse imaging data showed that within a 15 min timeframe, the clathrin-coated pits (CCPs) of *Cryba1* cKO RPE cells remained at the apical membrane without pinching off from the plasma membrane, while the CCPs in the floxed control cells remained at the apical membrane only briefly before internalization (Supplementary Movies 1 and 2 and Fig. 5b–d). To better depict this difference, we generated a kymograph of representative images from floxed control and *Cryba1* cKO samples, where solid, horizontal lines depict CCPs staying on the cell membrane (Fig. 5b). The data suggest that a significant number of CCPs in cKO RPE cells have a relatively long duration time at the apical membrane. We also employed highly inclined and laminated optical sheet (HiLo) microscopy to image the area beneath the apical surface. HiLo imaging also showed that movement of clathrin-coated vesicles was dynamic in floxed control RPE cells, but slower in *Cryba1* cKO RPE cells (Supplementary Movies 3 and 4 and Fig. 5e). In addition, fewer and larger clathrin-coated vesicles were observed in *Cryba1* cKO RPE cells compared to control RPE cells (Supplementary Movies 3 and 4 and Fig. 5f). The above observations suggest a critical role of βA3/A1-crystalin not only in clathrin-mediated EGFR endocytosis, but also in the membrane trafficking dynamics of polarized RPE cells.

To determine if the CCPs that failed to pinch off from the plasma membrane were properly loaded with the cargo (EGF) and if EGFR internalization was hindered in the RPE due to loss of βA3/A1-crystallin, RPE flatmounts from *Cryba1* cKO mice were infected with Ad-CMV-m*Clta*-FAP and rAV-CMV-LifeAct-TagGFP2, stimulated with Alexa-Fluo 555 EGF for 2 min after serum starvation, and fixed at 2, 5, and 10 min after EGF stimulation. Using TIRF microscopy, the number of EGF-loaded CCPs at the apical side gradually decreased over the tracking time in control RPE cells (Fig. 5g). However, a significant number of EGF-loaded CCPs remained at the apical side of *Cryba1* cKO RPE cells at 10 min post EGF stimulation, suggesting a failure of EGF-loaded CCPs to pinch off from the cell membrane of *Cryba1* cKO RPE cells (Fig. 5g). We also separated membrane fractions from lysates of the RPE flatmounts from control and cKO mice with or without EGF stimulation and detected EGFR in these fractions (Fig. 5h). We observed decreased membrane EGFR after 30 min of EGF stimulation in control RPE flatmounts. However, we did not observe significant reduction of membrane EGFR in *Cryba1* cKO RPE flatmounts. These data indicate that less EGFR was degraded in cKO RPE cells compared to the control, and further imply that the defective EGFR internalization might lead to less degradation in the RPE lacking βA3/A1-crystallin.

Interestingly, we found that a phosphorylation proteomics experiment we had performed previously showed some abnormally phosphorylated endocytosis-related proteins, such as formin-binding protein 17 (FNBP1) and amphiphysin II (BIN1), which are key mediators in CCP fission^48^ in *Cryba1* cKO RPE cells (Supplementary Table 1). We wondered if insufficient CCP fission was the rate-limiting factor preventing CCP pinching off from the plasma membrane in *Cryba1* cKO RPE cells. Therefore, we measured the FNBP1 activity during clathrin-mediated endocytosis in control and cKO RPE flatmounts. RPE flatmounts from *Cryba1* cKO and floxed control mice were infected with rAV-CMV-LifeAct-TagGFP2, Ad-CMV-FAP-m*Clta*, and Ad-CMV-m*Fnbp1*, and stimulated with EGF. With time-lapse imaging by TIRF microscopy, CCPs that were trapped at the apical membrane surface co-localized with FNBP1 in *Cryba1* cKO RPE cells, indicating defective fission in RPE cells of *Cryba1* cKO mice (Supplementary Movies 5 and 6 and Supplementary Fig. 3c, d). We speculate that the defective EGFR internalization may be due to the insufficient CCP fission. How βA3/A1-crystallin regulates EGFR activation and internalization remains to be determined.

### βA3/A1-crystallin interacts with PITPβ and may regulate PIP levels in RPE cells

**Fig. 6:**
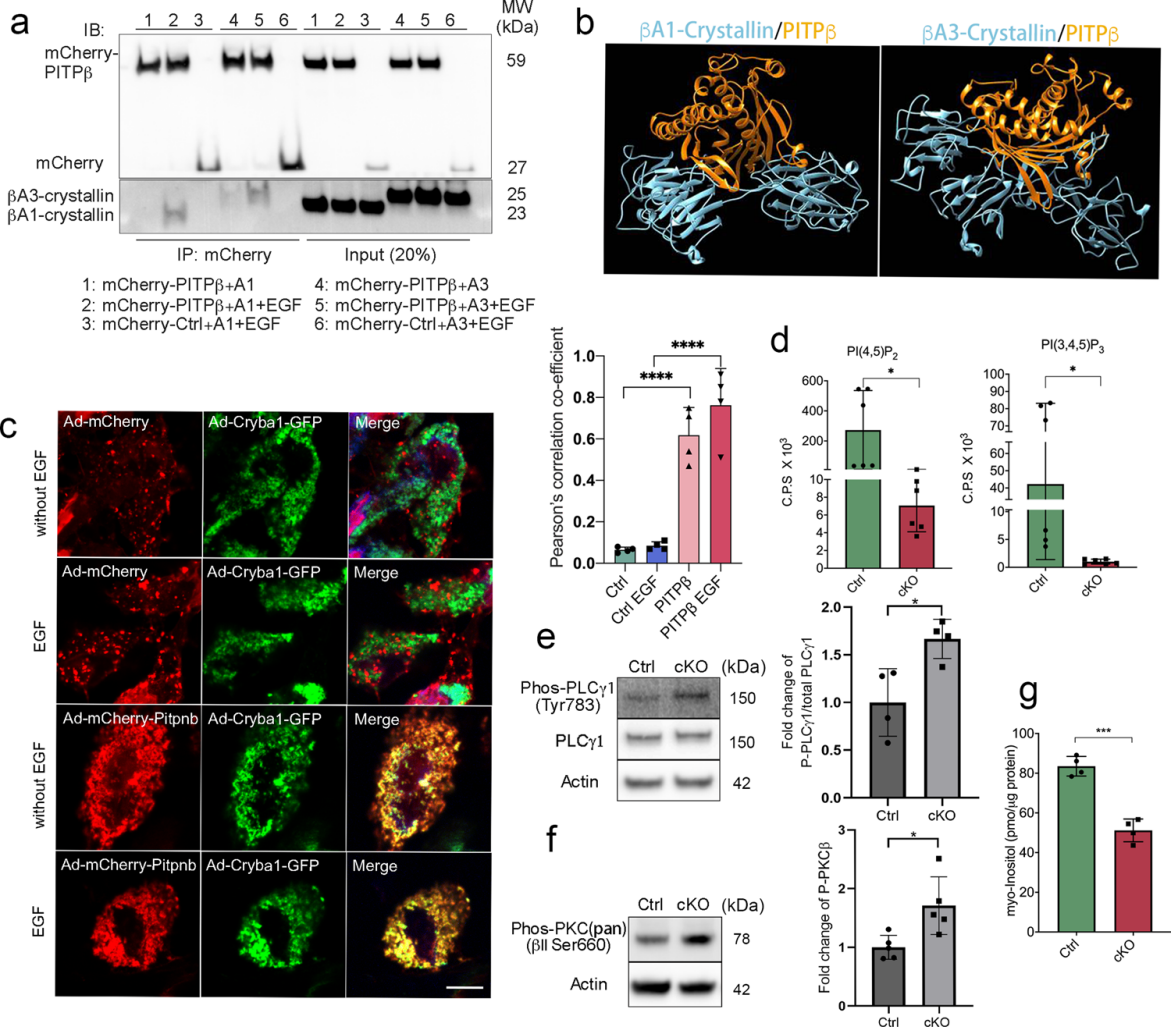
βA3/A1-crystallin interacts with PITPβ and may regulate PIP levels in RPE cells. **a** RPE flatmounts isolated from wildtype mice were cultured and infected either with Ad-mCherry-*Pitpnb* or Ad-mCherry-control and Ad-*Cryba1*-A3 or Ad-*Cryba1*-A1, respectively. RPE flatmounts were starved in FBS-free medium for 2 h with or without 30 min of EGF stimulation. RPE cell lysates were subjected to co-immunoprecipitation using RFP magnetic beads and the immunoprecipitants were analyzed by western blotting. Data show interaction of PITPβ with βA3/A1-crystallin, especially during EGF stimulation. **b** Protein complexes of βA3-crystallin/PITPβ and βA1-crystallin/PITPβ as predicted by molecular modeling. βA1-crystallin (left) and βA3-crystallin (right) are shown in blue. PITPβ is shown in orange. Both βA3- and βA1-crystallins are represented as the dimers formed by two “open” conformers. **c**. ARPE19 cells were infected with Ad-mCherry-*Pitpnb* or Ad-mCherry-control and Ad-*Cryba1*-GFP. Ad-*Cryba1*-GFP vector contains the wildtype *Cryba1* gene, and produces both βA3-crystallin and βA1-crystallin. Cells were treated with or without 10 ng/mL EGF for 10 min and fixed for imaging and colocalization analysis. Data show the colocalization of PITPβ and βA3/A1-crystallin, especially during EGF stimulation. Scale bar: 10 μm. **d** PI(4,5)P_2_ and PI(3,4,5)P_3_ were analyzed on RPE–choroid complexes of 2-month-old control and cKO mice. Data indicate a markedly reduced amount of PI(4,5)P_2_ and PI(3,4,5)P_3_ in cKO RPE–choroid complexes compared to that of control. *n* = 6. **e** Phosphorylated PLCγ1 levels were determined in RPE lysates of 3-week-old control and cKO mice by western blot. Data suggest increased PLCγ1 activity in cKO RPE cells. *n* = 4. **f** Phosphorylated PKC (pan) (βII Ser660) levels were determined in RPE lysates of 3-week-old control and cKO mice. Data suggest the activation of PKC in cKO RPE cells. *n* = 4. **g** Myo-inositol was analyzed on RPE–choroid complexes of 2-month-old control and cKO mice. Data indicate reduced myo-inositol level in cKO RPE–choroid complexes compared to that of control. *n* = 4. Statistical analysis was performed using two-tailed unpaired Student’s *t*-test (**d**–**g**) and one-way ANOVA (**c**). *P < 0.05, ***P < 0.001, ****P < 0.0001.

Having demonstrated that βA3/A1-crystallin has a role in the regulation of the apical cytoskeleton and in EGFR endocytosis, we investigated in our human proteome high-throughput array for upstream regulators that could interact with βA3/A1-crystallin to initiate these effects, and identified a very significant binding signal between βA3/A1-crystallin and PITPβ (phosphatidylinositol transfer protein beta isoform)^23^. PITPβ catalyzes the transfer of phosphatidylinositol and phosphatidylcholine between membranes and participates in PL metabolism during signal transduction and vesicular trafficking^49^. As mentioned above, a single *Cryba1* mRNA uses alternative translation start sites to produce βA3 and βA1-crystallin isoforms by leaky ribosomal scanning^28^. Since we identified multiple functions of this protein in RPE cells, and we have also previously found that βA3- and βA1-crystallins have different roles in astrocytes^50^, we wondered if the binding to PITPβ was isoform-specific. Using a co-immunoprecipitation assay on mouse RPE flatmounts overexpressing PITPβ and either the βA1- or βA3-crystallin isoform by adenovirus constructs, we confirmed that PITPβ binds to both isoforms (Fig. 6a). The binding was increased after EGF stimulation. A significant colocalization of PITPβ and βA3/A1-crystallin was also observed in ARPE19 cells overexpressing PITPβ and βA3/A1-crystallin either with or without EGF stimulation (Fig. 6c). Moreover, molecular modeling predicted an interaction of PITPβ with either βA3- and βA1-crystallin (Fig. 6b), with βA1 likely the better docking isoform.

One of the main functions of PITPβ in cellular signaling is to present phosphatidylinositol to lipid kinases for localized production of PI(4,5)P_2_^51^. Therefore, we measured PI(4,5)P_2_ in floxed control and cKO RPE cells. PI(4,5)P_2_ is the dominant phosphoinositide at the cell plasma membrane and the most important phosphoinositide for endocytosis^52^. Interestingly, we found a significantly decreased level of PI(4,5)P_2_ in *Cryba1* cKO RPE compared to control RPE (Fig. 6d). It has been reported that PITPβ and other PITPs enhance phospholipase C (PLC) activity, which mediates PI(4,5)P_2_ hydrolysis^53–55^. Notably, a recent study indicates that Sec14l3, a PITP family member, regulates the PI(4,5)P_2_ level by modulating PLC activation by a mechanism not involving PI transfer activity^54^. Therefore, we postulated that βA3/A1-crystallin may affect PI(4,5)P_2_ levels by influencing PLC activity. As expected, phosphorylated PLCγ1, which catalyzes the reaction of PI(4,5)P_2_ into second messengers inositol triphosphate (IP3) and diacylglycerol (DAG), was upregulated in *Cryba1* cKO RPE (Fig. 6e), indicating enhanced PI(4,5)P_2_ hydrolysis in cKO RPE cells. IP3 and DAG, the hydrolysis products of PI(4,5)P_2_, activate protein kinase C (PKC)^56^. Using the phospho-PKC (pan) (βII Ser660) antibody that detects PKC α, β I, β II, δ, ε, η, and θ isoforms only when phosphorylated at a carboxyl-terminal residue homologous to serine 660 of PKC β II, we found that PKC was significantly phosphorylated in *Cryba1* cKO RPE (Fig. 6f), consistent with the decreased levels of PI(4,5)P_2_ observed. We also found significantly reduced levels of phosphatidylinositol (3,4,5)-trisphosphate (PI(3,4,5)P_3_) in *Cryba1* cKO RPE compared to control cells. This may be due to reduced levels of PI(4,5)P_2_, since PI(4,5)P_2_ serves as a substrate for phosphoinositide 3-kinase (PI3K) when generating PI(3,4,5)P_3_^7^. The decrease in PI(3,4,5)P_3_ in *Cryba1* cKO RPE could also be due to reduced EGFR activation in *Cryba1* cKO RPE (Fig. 2b) because PI3K can be activated by phosphorylated EGFR^57^. It has been well-documented that PLC interacts with EGFR and relays signals from activated EGFR^58^. However, it has also been reported that PKC, the downstream effector of PLC, counteracts EGFR dimerization, acting as a negative feedback regulator of EGFR signaling^59^. Here, we found that in the absence of βA3/A1-crystallin, PLCγ1 signaling is activated while EGFR activation is compromised, which indicates that the βA3/A1-crystallin/PLCγ/PKC axis may play a pivotal role in EGFR activation at the apical surface of RPE cells.

Moreover, we observed that myo-inositol, an important component of PI and PIPs^60^, was also lower in *Cryba1* cKO RPE–choroid samples compared to controls, indicating an essential role of βA3/A1-crystallin in maintaining the PI(4,5)P_2_ pool (Fig. 6g). It is known that PI(4,5)P_2_ participates in CCP assembly and fission, activating EGFR, and potentiating its subsequent internalization^48,61^. We propose that the compromised EGFR activation and the defects in clathrin-coated structures in *Cryba1* cKO RPE cells are due to decreased PI(4,5)P_2_. Since PI(4,5)P_2_ is required for activating ezrin to link microfilaments to the plasma membrane^4^, the loss of PI(4,5)P_2_ could also induce microvilli disorganization observed in *Cryba1* cKO RPE cells.

### βA3/A1-crystallin attenuates PLCγ signaling, thereby activating ezrin phosphorylation and the EGFR signaling network

**Fig. 7:**
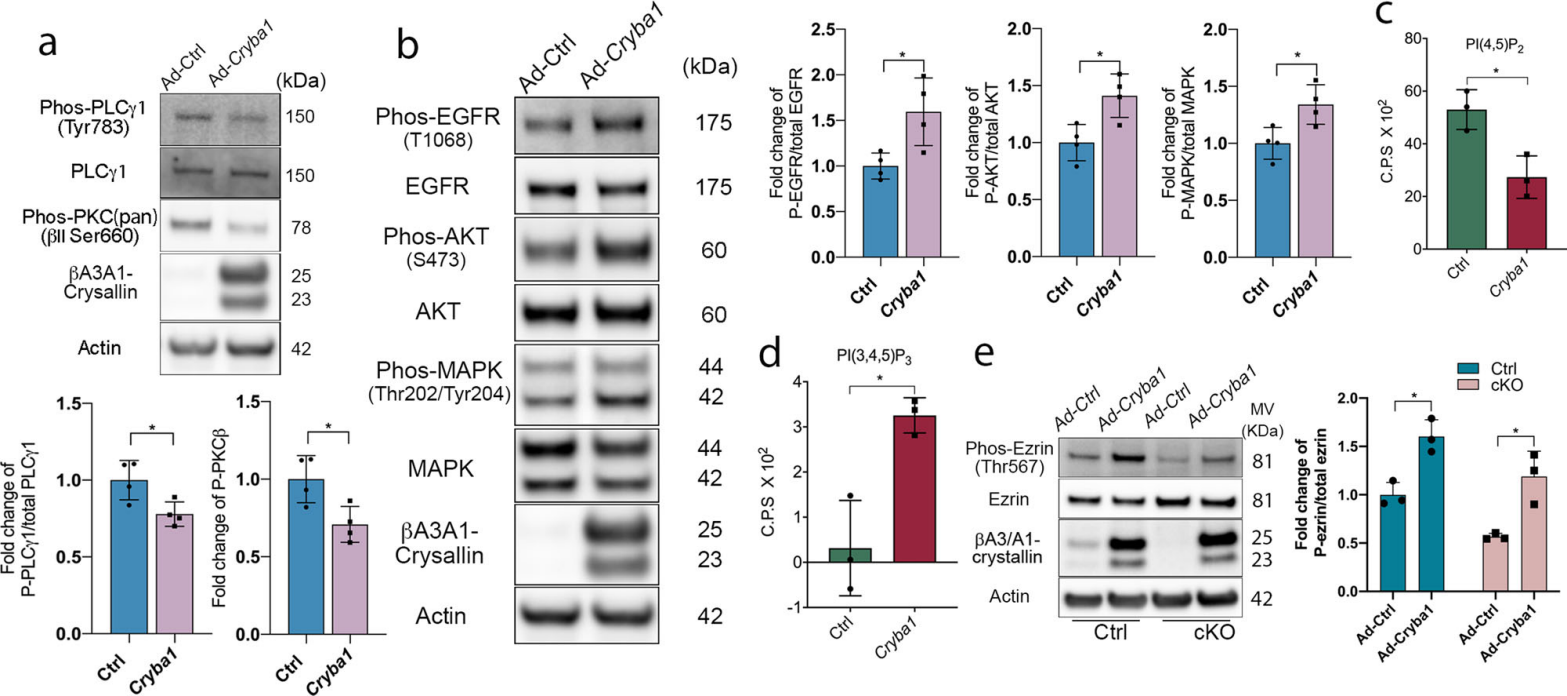
βA3/A1-crystallin attenuates PLCγ signaling and activates ezrin phosphorylation and the EGFR signaling network. **a** RPE flatmounts from 2-month-old *Cryba1* cKO mice were cultured and infected with either Ad-RFP-m*Cryba1* or Ad-CMV-RFP control viral vector. Western blot analysis was performed on lysates to detect PLCγ1 and PKC activity. Graphs show reduced PLCγ1 and PKC phosphorylation in *Cryba1* cKO RPE cells overexpressing *Cryba1*. *n* = 4. **b** RPE flatmounts from 5-month-old *Cryba1* cKO mice were cultured and infected with either Ad-RFP-m*Cryba1* or Ad-CMV-RFP control viral vectors. Flatmounts were stimulated with 50 ng/mL EGF for 60 min after serum starvation. Cells were then lysed and western blot analysis was performed on the lysates to detect EGFR phosphorylation and its downstream signaling molecules. Upregulated phosphorylation of EGFR, AKT1, and MAPK was observed in *Cryba1* cKO RPE cells overexpressing *Cryba1*. *n* = 4. **c**, **d** RPE flatmounts from 2-month-old *Cryba1* cKO mice were cultured and infected with either Ad-RFP-m*Cryba1* or Ad-CMV-RFP control viral vector. PI(4,5)P_2_ (**c**) and PI(3,4,5)P_3_ (**d**) levels were analyzed. *n* = 3. Data showed increased PI(3,4,5)P_3_ but decreased PI(4,5)P_2_. **e** RPE flatmounts from 2-month-old *Cryba1* cKO mice and control mice were cultured and infected with either Ad-RFP-m*Cryba1* or Ad-CMV-RFP control viral vector. Western blot analysis was performed on lysates to detect ezrin phosphorylation. Graph shows increased phosphorylation of ezrin in both control and *Cryba1* cKO RPE cells overexpressing *Cryba1*. *n* = 3. The error bars represent the SD of the mean. The statistical analysis was performed using two-tailed unpaired Student’s *t*-test (**a**–**d**) and two-way ANOVA (**e**). *P < 0.05.

In order to confirm our findings that βA3/A1-crystallin regulates PI(4,5)P_2_ and PI (3,4,5)P_3_ levels and is involved in the EGFR signaling network, we overexpressed βA3/A1-crystallin in *Cryba1* cKO RPE flatmounts using a *Cryba1* adenovirus construct. We found that phosphorylated PLCγ1 and PKC were decreased in *Cryba1* cKO RPE cells with *Cryba1* overexpression (Fig. 7a). Importantly, when stimulated by EGF, EGFR phosphorylation was significantly upregulated in RPE cells with *Cryba1* overexpression compared to the cells infected with control constructs. The EGFR downstream effectors AKT and MAPK were also activated after *Cryba1* overexpression (Fig. 7b). These data confirmed that βA3/A1-crystallin promotes EGFR activation and its downstream signaling pathways. In RPE flatmounts with either *Cryba1* or control expression vectors, we found that the PI(3,4,5)P_3_ level was significantly increased when *Cryba1* was overexpressed (Fig. 7d). However, unexpectedly, the PI(4,5)P_2_ level was decreased in cKO RPE cells overexpressing βA3/A1-crystallin compared to that in cKO RPE cells with control vectors (Fig. 7c). It is possible that the PI(4,5)P_2_ pool is highly dynamic and the EGFR/PI3K/AKT signaling pathway is activated after *Cryba1* overexpression, and that more PI(4,5)P_2_ is converted to PI(3,4,5)P_3_, accounting for the decrease in PI(4,5)P_2_. In addition, both control and cKO RPE flatmounts have elevated ezrin phosphorylation levels following *Cryba1* overexpression (Fig. 7e). Overall, our data demonstrate that βA3/A1-crystallin regulates PIP levels by modulating PLCγ activity, thus activating EGFR signaling and ezrin phosphorylation in RPE cells.

## Discussion

In this study we made the unique observation that in RPE cells, βA3/A1-crystallin regulates PIP metabolism, most likely by binding to PITPβ, a protein that transfers phosphatidylinositol and phosphatidylcholine between membranes, and presents phosphatidylinositol to lipid kinases for localized production of PI(4,5)P_2_. Mutations in PITPβ or other PITP proteins such as rdgB (retinal degeneration B) have been shown to result in retinal degeneration in both invertebrate and vertebrate models^62–66^.

In RPE lacking βA3/A1-crystallin, we found decreased PI(4,5)P_2_ levels and increased activity of phospholipase C, the enzyme that hydrolyzes PI (4,5)P2^58^. In addition, upon overexpression of βA3/A1-crystallin in RPE flatmounts, a reduction in PLC/PKC activity was observed. We speculate that βA3/A1-crystallin regulates PLC activity by influencing PITPβ activity. Previously, PITPs were shown to enhance PLC activity^53–55^. We also postulate that PI(4,5)P_2_ levels are responsive to βA3/A1-crystallin through the PITPβ/PLC axis. While dysregulation of PIP metabolism in photoreceptors has been reported to cause blindness^8–12^, the roles of PIPs in RPE function and physiology remain uncertain.

**Fig. 8:**
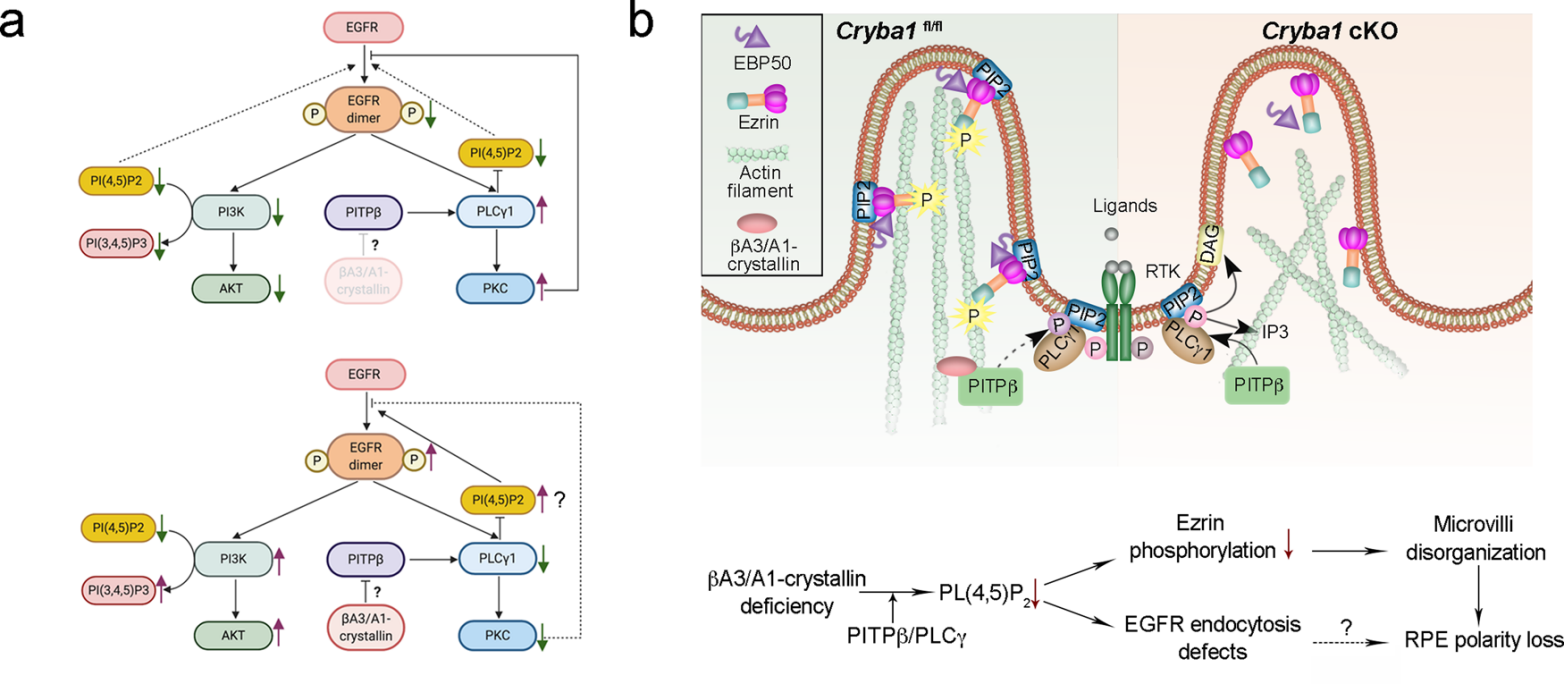
The possible role of βA3/A1-crystallin in the EGFR signaling network and RPE polarity. **a** A model showing the possible role of βA3/A1-crystallin in the PITPβ/PLCγ signaling axis and PI(4,5)P_2_ and PI(3,4,5)P_3_ turnover, and consequently in EGFR activation and internalization. Top: RPE cells lacking βA3/A1-crystallin have increased PLCγ1 and PKC activity, which leads to decreased PI(4,5)P_2_. EGFR activation is inhibited by the PI(4,5)P_2_ deficiency and activated PKC through a negative feedback loop. Bottom: overexpression of βA3/A1-crystallin attenuated phosphorylated PLCγ1, possibly by influencing PITPβ activity. The PI(4,5)P_2_ level may be temporarily increased. Having sufficient PI(4,5)P_2_ promotes EGFR activation. On the other hand, decreased PKC activity may not affect EGFR dimerization. Due to the activation of EGFR, more PI(4,5)P_2_ was converted to PI(3,4,5)P_3_ because of the activation of AKT by PI3K, which may subsequently lead to PI(3,4,5)P_3_ upregulation and PI(4,5)P_2_ downregulation. **b** Cartoon depicting the pathway initiated by the βA3/A1-crystallin deficiency leading to RPE apical surface disruption and impaired EGFR endocytosis. Loss of functional βA3/A1-crystallin prevents its interaction with PITPβ causing a modification of PIP metabolism that eventually impairs both EGFR endocytosis and ezrin phosphorylation, thereby altering the actin cytoskeleton and affecting RPE cell polarity.

PI(4,5)P_2_ is the most abundant phosphoinositide at the plasma membrane, and it binds to proteins that are required for actin filament assembly (e.g., ezrin/radixin/moesin, profiling, cofilin, etc.) and endocytosis (e.g., picalm, dynamin, epsin, etc.)^67^. We propose that PI(4,5)P_2_ is essential for maintaining the RPE apical cytoskeletal network and normal endocytosis. PI(4,5)P_2_ is the most important phosphoinositide for endocytosis;^52^ it is required for EGFR activation and clathrin-mediated endocytosis^61^. Indeed, we found compromised EGFR activation and internalization following EGF stimulation in RPE cells that lack βA3/A1-crystallin. With TIRF imaging of RPE flatmounts, we observed impaired CCP fission and EGFR internalization. Importantly, the defective EGFR activation in *Cryba1* cKO RPE cells can be rescued by overexpressing βA3/A1-crystallin. Interestingly, while a βA3/A1-crystallin deficiency altered the phosphorylation of EGFR (and the related ErbB2), it did not affect other RTKs. We speculate that during evolution, βA3/A1-crystallin obtained the specific ability to regulate EGFR signaling in RPE cells, and that the molecular coordination of this signaling pathway is unique to RPE cells. This may be analogous to the recent postulation that the RPE has a specialized and unique clearance machinery that differs from traditional phagocytic cells^68^. The possible role of βA3/A1-crystallin in the EGFR signaling network is demonstrated in Fig. 8a. Our data showed that a βA3/A1-crystallin deficiency in RPE cells led to increased PLCγ1/PKC activity, thus decreased PI(4,5)P_2_ and EGFR activation, whereas βA3/A1-crystallin overexpression inhibited phosphorylation of PLCγ1, probably resulting in a rapid increase in PI(4,5)P_2_ levels, which led to EGFR activation. Meanwhile, the decreased PKC activity in the presence of βA3/A1-crystallin might disrupt the negative feedback effect on EGFR dimerization. Due to activated EGFR, the conversion of PI(4,5)P_2_ to PI(3,4,5)P_3_ was enhanced because of AKT activation by PI3K (Fig. 8a). Interestingly, it was previously reported that PITP is required for EGFR signaling and that it can bind to EGFR and PLCγ in response to EGF stimulation. Therefore, we propose here that βA3/A1-crystallin modulates EGFR activation by influencing PITPβ/PLCγ activity (Fig. 8a). The EGFR activation defects might also contribute to RPE polarity loss and RPE degeneration; however, this possibility needs to be investigated in the future (Fig. 8b).

Furthermore, PI(4,5)P2 is known to bind to ezrin, a member of the ezrin/radixin/moesin (ERM) family of proteins. ERM proteins provide a critical link between the plasma membrane and the underlying actin cytoskeleton. Ezrin is highly expressed in epithelial cells that have microvilli, such as RPE. Its phosphorylation is regulated by PI(4,5)P_2_, and it is essential for establishing microvilli and maintaining polarity^4^. Here, we show that βA3/A1-crystallin is required by RPE cells to maintain normal ezrin phosphorylation and polarity. When βA3/A1-crystallin is absent, PI(4,5)P_2_ and ezrin phosphorylation are also substantially reduced, and interaction between ezrin and the cytoskeleton is disrupted. As a result, morphological and functional changes at the apical surface are altered, and RPE cells lose polarity (Fig. 8b). In this study, we provide direct evidence that βA3/A1-crystalllin affects microvilli organization by modulating PI(4,5)P_2_ and ezrin phosphorylation.

These changes are highly relevant to AMD, the most common cause of blindness among the elderly in Western societies^69^. Rod photoreceptor death secondary to RPE degeneration in the perifoveal macula is one of the earliest changes in AMD^70^, and loss of apical microvilli is an early sign of this process^5^. With a similar RPE and rod/cone density as the human perifovea, mice are a reasonable model for studying early AMD changes^71^. Thus, the apical microvilli changes in the RPE of *Cryba1* cKO mice, an established model of early perifoveal AMD-like pathobiology, provide us with new avenues for investigating both how the RPE loses its polarity, especially in the apex, and the impact that this loss has on the health of both the RPE and the overlying photoreceptors during disease onset and progression. Interestingly, loss of apical processes is considered integral to the loss of RPE polarity in atrophic AMD. Immunohistochemistry studies have shown decreased expression of ezrin in the macula of geographic atrophy eyes^72^. By utilizing this unique animal model, we identified the role of βA3/A1-crystallin in regulating two processes most likely through modulating PI(4,5)P_2_ pool at the apical side of RPE: ezrin’s interaction with the actin cytoskeleton and membrane trafficking during EGFR endocytosis, which are absolutely essential for maintaining RPE cellular polarity. We previously reported the dysfunction of the retina in *Cryba1* cKO mice; assessment included fundus imaging and electroretinogram measurements^24^. We can only speculate that the retina dysfunction is partially caused by the RPE polarity loss, since the lysosomal function is also impaired in the RPE cells in the *Cryba1* cKO model. In this study, we focus on the function of βA3/A1-crystallin in RPE polarity and the molecular mechanism underlying this regulation. It is worth investigating the detailed consequences of RPE polarity disruption on retinal function in *Cryba1* cKO, but it is beyond the scope of the current study. Taken together, our data suggest a pathway initiated by a βA3/A1-crystallin deficiency that explains how apical microvilli become disorganized and how EGFR endocytosis is impaired during RPE degeneration in a model that is highly relevant to early AMD.

Finally, crystallins are intriguing for their multifunctionality in non-lens tissues as moonlighting proteins^17^. We have previously shown that βA3/A1-crystallin is localized to the lysosomal lumen in RPE cells, which is required for autophagy and phagocytosis^21,24^. In this study, to the best of our knowledge, we, for the first time, identified the functions of βA3/A1-crystallin in the apical region of polarized RPE cells, including its regulation of PIP metabolism through interaction with PITPβ, its role in EGFR signaling, and its role in ezrin activation and microvilli organization. We postulate that βA3/A1-crystallin may regulate diverse subcellular processes as a moonlighting protein. The binding of βA3/A1-crystallin and PITPβ indicates its location in the Golgi and endoplasmic reticulum (ER). It is known that newly synthesized lysosomal proteins are first transferred into the ER lumen, followed by the Golgi apparatus and are then transported from the trans Golgi network to late endosomes. This is consistent with the βA3/A1-crystallin protein being localized to the ER and Golgi at some point of its existence. In addition, Ma et al. demonstrated that βA3/A1-crystallin is required for trafficking the anoikis regulator, Bit1, to the Golgi in the astrocytes, thus putting this protein’s function in the context of processes that take place in the Golgi apparatus.

It is worth mentioning that *Cryba1* is unusual among eukaryotic genes in producing two proteins by leaky ribosomal scanning. In eukaryotes, genes with leaky ribosomal scanning produce functionally important protein isoforms with distinct roles^73^. When translation is initiated using alternative start sites (βA3-crystallin has 17 additional N-terminal amino acid residues), it is predicted that the isoforms are delivered to different subcellular compartments^74,75^. While our studies to date do not address whether βA3- and βA1-crystallins are functionally different in RPE cells, we have shown βA3/A1-crystallin in different compartments of RPE cells and astrocytes^23,24,50,76^. In future studies, we intend to determine whether the βA3- and βA1-crystallin isoforms possess different properties and functions at different subcellular locations.

## Methods

### Animals

βA3/A1‐crystallin conditional knockout (*Cryba1* cKO) mice and control *Cryba1*^fl/fl^ mice were generated as previously described^24^. All animal studies were conducted in accordance with the Guide for the Care and Use of Animals (National Academy Press) and were approved by the Animal Care and Use Committee of the University of Pittsburgh.

### Immunostaining of retina cryosections and RPE flatmounts

Fresh eyes were enucleated and fixed in 2% paraformaldehyde (PFA) for 10 min followed by the removal of the anterior parts including cornea, lens, and the attached iris pigmented epithelium. The resulting posterior eyecups were fixed in 2% PFA for 1 h at room temperature either for cryosections or RPE flatmounts. For the retina cryosections, the eyecups were dehydrated using gradient sucrose solutions and embedded in OCT compound for sectioning. For the RPE flatmounts, the eyecups were quartered like a petaloid structure and the retinas were removed. The resulting RCS complexes or retina cryosections were permeabilized with 0.25% Triton X-100 in PBS for 15 and 10 min, respectively. RCS complexes or retina sections were blocked in blocking buffer (2% goat serum, 1% BSA, and 0.1% Triton X-100 in PBS) for 30 min at room temperature followed by incubation in EBP50 antibody (1:200, PA1090, Thermo Fisher Scientific) in blocking buffer in a humidified chamber overnight at 4 °C. RCS complexes or retina sections were then washed with 1x PBS and incubated with the secondary antibody (1:200, Goat anti-Rabbit, Alexa Fluor 568, A-11011, Invitrogen), Alexa Fluor 488 Phalloidin (1 unit/mL, A12379, Invitrogen), and 1 μg/mL DAPI (D1306, Thermo Fisher) in blocking buffer for 1 h in the dark at room temperature. After washing, RCS complexes were flattened on a microscope slide with the RPE layer up. Retina sections or RCS complexes were then mounted with DAKO mounting medium (S3023, Agilent) and sealed with a coverslip. Images were acquired on a Zeiss LSM 710 confocal workstation. Microvilli height was measured by the length measurement tool available in ZEN software (Zeiss, Germany) based on the staining results. Other antibodies used for immunostaining: MCT3 (1:200, AMT013, Alomone Labs), Sodium Potassium ATPase, (1:200, ab76020, Abcam), and β-catenin (1:200, ab16051, Abcam).

### Transmission electron microscopy

Samples were prepared and transmission electron microscopy was performed as previously described^21^. Briefly, mouse eyes were enucleated immediately after CO_2_ euthanasia; a small slit was made at the limbus and then fixative (2.5% glutaraldehyde with 2% PFA in 0.1 mol/L cacodylate buffer, pH 7.4) was injected into the globe. Each eye was then immersed in the same fixative solution. Anterior segments were removed and the resulting eyecups were washed in 0.05 mol/lL cacodylate buffer and post-fixed in 1% OsO_4_ in 0.05 mol/L cacodylate buffer for 90 min. The eyecups were then washed with 0.05% cacodylate buffer and dehydrated in a graded series of ethyl alcohol and stained en bloc with 1% uranyl acetate in 100% ethanol followed by two 15 min incubations in propylene oxide. The tissue was then kept overnight in a 1:1 propylene oxide and resin mixture. The tissue was then infiltrated in 100% LX112 resin (Ladd Research Industry, Burlington, VT) for 4–6 h under vacuum, embedded in a final change of 100% LX112 resin and then polymerized at 60 °C for 36–48 h. Ultrathin sections were cut with a Leica Ultramicrotome UCT (Leica Microsystems, Wetzlar, Germany), stained with uranyl acetate and lead citrate and visualized with an H7600 transmission electron microscope (Hitachi, Tokyo, Japan).

### Tissue collection and RPE protein preparation

Eyeballs were enucleated from euthanized mice, and anterior cornea, lens, and neural retina were removed. The resulting RCS complexes were snap-frozen on dry ice. RPE cells were mechanically isolated from RCS complexes by tapping for 30 s in RIPA lysis buffer (20-188, Millipore) with a mixed protease and phosphatase inhibitor cocktail as described^77^. Choroid-sclera complexes were then removed from the lysate buffer. The resulting RPE cells were sonicated for 15 s on ice and incubated in lysis buffer for 15 min. RPE lysates were separated from the cell debris by centrifugation at 15,000 rpm for 20 min. The protein concentrations were determined by BCA assay.

### Phospho-RTK array

Mice were fasted for 36 h by withholding food without restriction on water availability, with or without a subsequent 3 h refeeding. All mice were sacrificed between 10 am and 12 pm. The RPE lysates were extracted as described above. Each RPE sample was pooled from four mice that had received the same treatment. An equal amount of each sample was used to quantify phospho-RTK proteins with a proteome profiler mouse phospho-RTK array kit (ARY014, R&D Systems). Quantification of the spot intensity in the arrays was conducted with Quantity One software (Bio-Rad).

### RPE flatmount culture and EGF stimulation

Eyeballs were enucleated from 2-month-old mice immediately after euthanasia, and the anterior segments were removed thereafter. The resulting posterior eyecups were cut into four petals and the neural retina was carefully removed. The resulting RCS complexes were flattened onto PVDF membranes with the RPE cells facing up and cultured in a 37 °C incubator in complete Dulbecco Modified Eagle Medium (DMEM), high glucose, supplemented with 2% FBS, 1% penicillin/streptomycin, 2.5 mM L-glutamine, and 1x MEM nonessential amino acids. The mouse HPV E6/E7 RPE cells (T0574, ABM) were cultured in Prigrow III Medium (TM003, ABM) with 10% FBS and 1% penicillin/streptomycin. Before EGF stimulation, RPE flatmounts/mouse HPV E6/E7 RPE cells were starved in FBS-free medium for 2 h. RPE flatmounts/mouse HPV E6/E7 RPE cells were then cultured in DMEM medium containing 50 ng/mL EGF (AF-100-15, Peprotech) for 5, 15, 30, and 60 min, respectively. RPE flatmounts were then immediately washed with PBS and snap-frozen on dry ice. RPE lysates were isolated from flatmounts as described above. Mouse HPV E6/E7 RPE cells were washed in PBS and collected for western blot analysis. For the positive control of cell death, RPE flatmounts were cultured in complete medium containing 10 and 100 μM H_2_O_2_ for 16 h. RPE cell lysates were collected for western blot analysis.

### EGFR endocytosis analysis by total internal reflection fluorescence (TIRF) microscopy and highly inclined and laminated optical sheet (HiLo) microscopy

For endocytosis analysis in live RPE cells, RPE flatmounts were cultured in complete medium in a glass-bottom dish and infected with recombinant human serotype 5 adenoviral constructs with deleted E1/E3. Ad-CMV-FAP-m*Clta* is a CMV promoter-driven vector that contains mouse *Clta* cDNA and a FAP reporter. rAV-CMV-LifeAct-TagGFP2 (60121, ibidi) was used for labeling the F-actin network. Ad-CMV-m*Fnbp1* was made by inserting the cloned mouse Fnbp1 cDNA into the recombinant human serotype 5 adenoviral constructs. The flatmounts were infected at 5 × 10^6^ PFU/mL of each adenovirus for 12–16 h. The RPE flatmounts were then starved in DMEM medium containing a cell-permeable FAP fluorogenic dye (MG-ester, 50 nM) for 2 h. The starvation medium was replaced with 50 ng/mL EGF containing medium immediately and the flatmounts were placed apical surface down against the coverslip of the glass-bottom dish and is gently compressed such that the apical surface enters the TIRF plane. The device consists of a 3D printed black lid that clips onto the imaging dish and a plunger that is axially positioned using a fine pitch thread. As the plunger was screwed down, the RPE flatmount (mounted on a non-moving, bearing mounted plate at the end of the plunger) came into contact with the coverslip and can be imaged. This was done on the microscope to ensure that there was no compressive damage to the cells as they were imaged. RPE flatmounts were immediately taken for TIRF time-lapse imaging. TIRF images of RPE flatmounts were taken on an inverted Nikon Ti with a Plan Apo TIRF 1.49 NA 100x oil objective and Prime 95b camera (Photometrics). Images were acquired with a 100 ms exposure time. All samples were imaged in a chamber at 37 °C with 5% CO_2_. Subsequent image analysis included a modified Richardson-Lucy deconvolution step and no other processing was performed. Kymographs of the images in maximum intensity projection were generated using NIS-Elements (Nikon). Duration analyses of CCPs were performed using Imaris software. CCPs with a duration time of less than 30 s were filtered out for the analysis. HiLo microscopy was conducted on the same microscope. Illumination used a 640 nm laser at an incident angle that allowed the lower ~0.5 μm of the cell to be imaged. Images were acquired with a 100 ms exposure time. All samples were imaged in a chamber 37 °C with 5% CO_2_. Clathrin structures were determined with the spot detection function to create binary masks of clathrin by NIS-Elements. Speed and count analysis of CCVs were generated using NIS-Elements software. For EGFR endocytosis tracking analysis, clathrin and F-actin labeled mouse RCS complexes were transferred to DMEM medium containing a cell-permeable FAP fluorogenic dye (MG-ester, 50 nM) for 2 h. RCS complexes from *Cryba1* cKO and control mice were then stimulated with 50 ng/mL Alexa Fluor 555 EGF complex (E35350, Thermo Fisher Scientific) for 2 min in an incubator and then immediately moved either to PBS for washing and for subsequent fixation in 2% PFA or to FBS/EGF-free DMEM medium to extend the culture for extra 3 or 8 min for tracking purpose. Imaging quantification was performed in NIS-Elements. Clathrin and EGF localization and quantification were determined with the spot detection function to create binary masks of clathrin and EGF signals.

### RPE membrane fraction assay

RPE flatmounts from 2-month-old *Cryba1* cKO and age-matched control mice were cultured and stimulated with 50 ng/mL EGF (as described above) for 30 min. RPE flatmounts were washed in PBS three times and immediately snap-frozen on dry ice. The RPE cells were collected in permeabilization buffer (89842, Thermo Fisher) as described above. Membrane and cytoplasmic protein fractions of RPE cells were obtained with the Mem-PER plus membrane protein extraction kit (89842, Thermo Fisher).

### Phospho-proteomics

RPE samples were collected from 8-month-old *Cryba1* cKO and age-matched control mice. Each sample was pooled from four mice from the same group. RPE samples were digested by trypsin and the tryptic peptides were labeled using the TMT-10 plex labeling kit and fractionated by UHPLC (Poochon Scientific). The phosphorylated peptides were enriched from the fractions of TMT-10 plex labeled peptide using a TiO2 column from Pierce and analyzed by LC/MS/MS. The LC/MS/MS analysis of samples was carried out using a Thermo Scientific Q-Exactive Hybrid Quadrupole-Orbitrap Mass Spectrometer and a Thermo Dionex UltiMate 3000 RSLCnano System. For protein identification, MS files were analyzed using the Thermo Proteome Discoverer 1.4.1 platform (Thermo Scientific, Bremen, Germany) for peptide identification and protein assembly. A database search against public mouse proteins obtained from the NCBI website was performed based on the SEQUEST and percolator algorithms through the Proteome Discoverer 1.4.1 platform. The maximum false peptide discovery rate was specified as 0.01.

### RFP pulldown experiment

To produce adenovirus constructs for the βA1 isoform, the first codon of *Cryba1* was mutated and a silent mutation was also introduced to prevent the binding and re-cutting of the sequence by gRNA after homology-directed repair. To produce the longer (βA3) isoform, CCACC was inserted immediately before the first start site to strengthen the Kozak consensus sequence, thereby making the first start codon stronger. The second mutation was introduced to disrupt the possible Kozak sequence that was located upstream of the A1 start codon. Wildtype mouse RPE flatmounts were infected with Ad-CMV-mCherry-*Pitpnb* (or Ad-CMV-mCherry-control) and either Ad-CMV-A3 or Ad-CMV-A1 for 16 h. RPE flatmounts were then stimulated with 50 ng/mL EGF for 30 min. RPE lysates were collected. Cell extracts were incubated with RFP magnetic beads (rtmak-20, Chromotek) for 2 h at 4 °C. Collected beads were washed with washing buffer (10 mM Tris/Cl pH 7.5, 150 mM NaCl, 0.5 mM EDTA, and 0.018% sodium azide) and eluted with 2x SDS-sample buffer. The eluted samples were analyzed in a SDS-PAGE gel.

### ARPE19 cell culture and colocalization analysis

ARPE19 cells were obtained from American Type Culture Collection (Manassas, VA), and cultured in DMEM/F12 medium supplemented with 10% FBS and 1% penicillin/streptomycin. Cells were infected with Ad-CMV-m*Cryba1*-GFP and either Ad-CMV-mCherry-*Pitpnb* or Ad-CMV-mCherry control constructs for 24 h. Cells were then starved in FBS-free medium for 2 h and stimulated with or without 10 ng/mL EGF for 10 min. The colocalization analysis for dual-color observations was performed by using Fiji ImageJ software.

### Molecular modeling

The structures of βA3-crystallin, βA1-crystallin, and PITPβ were built by homology modeling using Yasara (http://www.yasara.org/). The molecules were docked and the complex structure was energy minimized in ucsf chimera (https://www.cgl.ucsf.edu/chimera).

### Phosphoinositide extraction and measurement

RPE–choroid complexes were isolated from 2-month-old *Cryba1* cKO and age-matched control mice. Each sample was pooled from four mice from the same group. All mice were sacrificed between 10 am and 12 pm. Mouse RPE was homogenized in PBS, and 2:1 chloroform:methanol was added to extract the phosphatidylinositol (PI). The extraction was repeated with chloroform to remove bulk PLs (PE, phosphatidylethanolamine; PC, phosphatidylcholine; PS, phosphatidylserine). The chloroform layers were pooled into one tube, which represents the PLs (PE, PC, and PS). This fraction does not contain phosphoinositides. To the remaining solution, chloroform:methanol:HCl (2:4:0.1) was added to extract the PI. The procedure was repeated with chloroform: methanol:HCl. Then, the chloroform layers were pooled into a clean tube, which represents the PI. The PI pooled fractions were dried under nitrogen gas and the dried lipids were dissolved in 150 μL of 1:9 chloroform:methanol, and sonicated. We measured lipid phosphorus from the PI fractions according to the method described^78^ and converted the determined lipid phosphorus concentration into PL concentration. In the conversion factor, PL = lipid phosphorus × 24.0, an “average phospholipid” of molecular weight 744 (i.e., an equimolecular mixture of oleopalmityl lecithin and cephalin) is assumed. The PI sample was used for the measurement of PI (4,5)P_2_ and PI(3,4,5)P_3_. The phosphoinositide assay is based on the interaction of PI with phosphoinositide-binding proteins^79^. Specifically, we measured lipid phosphorous from the PI fraction and converted the quantified lipid phosphorous into PL. We have expressed the PLCδ-PH domain that specifically binds to PI(4,5)P2 and Grp1-PH domain that bind specifically with PI(3,4,5)P3 in the bacteria as a maltose-binding protein (MBP) fused with a rhodopsin-1D4 epitope. The expressed proteins were purified on an amylose affinity column chromatography. An equivalent amount of PL from different samples was coated onto 96-well ELISA plate, and the dried lipids were blocked with 3%BSA in PBS at room temperature for 2 h. The ELISA plate was incubated with MBP-PLCδ-PH-1D4 and MBP-Grp1-1D4 (0.25–0.5 μg/mL) fusion proteins overnight. The plate was washed, then incubated with the rhodopsin-1D4 antibody for 2 h at RT. After washing the plate, a mouse secondary antibody coupled to the HRPO enzyme was added and incubated for 60 min at RT. The plate was washed and the luminescence was measured using an ELISA plate reader. Relative luminescence units (or counts per second) of each PI was normalized to PL.

### Myo-inositol measurement

RPE–choroid complexes were isolated from 2-month-old *Cryba1* cKO and age-matched control mice. Each sample was pooled from four mice from the same group. All mice were sacrificed between 10 am and 12 pm. The myo-inositol concentration in the RPE samples was determined as described in the reference^80^. Specifically, the method is based on the oxidation of myo-inositol by NAD+-dependent myo-inositol dehydrogenase, coupled to reoxidation of NADH by iodonitrotetrazolium chloride and diaphorase. RPE–choroid complexes were homogenized in (16%, w/v) perchloric acid and centrifuged. All supernatants were neutralized with 2.0 M K_2_CO_3_ and centrifuged. The supernatants were then added to hexokinase reagent (200 mM Tris–HCl buffer, 400 mM adenosine triphosphate disodium (pH = 8.6), and 115 U/mL hexokinase). The mixtures were incubated at 37 °C for 90 min, and heated for 3 min to stop the reaction. Endogenous NADH and NADPH were then eliminated by adding 4.5 M HCl. Then, 3.0 M K_2_CO_3_ was added to the mixtures for neutralization. Then, samples were mixed with 210 mM triethanolamine hydrochloride–32 mM K_2_HPO_4_–KOH buffer (pH 8.6), 1.2% (v/v) Triton X-100, 10 mM β-NAD, 1.0 U/mL diaphorase, 0.1% (w/v) bovine serum albumin, and 60 μg/mL INT in a 96-well microplate. The absorbance of the solutions was measured at 492 nm before and after the reaction by adding 2.1 U/mL MIDH dissolved in 20 mM potassium phosphate buffer (pH 7.0) to each well for 30 min at room temperature. The myo-inositol content was calculated based on the increase in absorbance during the reaction.

### *Cryba1* overexpression in *Cryba1* cKO RPE flatmounts

An Ad-CMV-RFP-m*Cryba1* vector was made by inserting mouse *Cryba1* cDNA into a human type 5 (dE1/E3) adenoviral backbone, with a RFP under its own CMV promoter. *Cryba1* cKO or control RPE flatmounts were prepared and infected with either Ad-RFP-m*Cryba1* or Ad-CMV-RFP at a concentration of 1 × 10^7^ PFU/mL for 16 h. RPE flatmounts were then processed for subsequent analysis.

### Western blotting analysis and antibodies

The native protein samples were mixed with 4x protein LDS sample buffer (NP0007, Invitrogen) containing 10% 2-mercaptoethanol and heated at 100 °C for 10 min. Then, 8~12 μg of protein from each RPE sample was loaded onto a 4–12% Bis-Tris Nu-PAGE gel (NP0323BOX, Invitrogen) and transferred to a nitrocellulose membrane. Membranes were blocked in either 5% non-fat milk (170-6404, Bio-Rad) or 5% BSA (A3912-50G, Sigma Aldrich), and then incubated with primary antibodies overnight at 4 °C followed by three washes (30 min total) and incubation with the appropriate HRP-conjugated secondary antibody for 1 h at room temperature. Blots were visualized using the ECL western blotting detection reagent (RPN2209, GE Healthcare Life Sciences). The following antibodies were used: phospho-Ezrin (Thr567) (1:1000, PA537763, Thermo Fisher Scientific), Ezrin (1:1000, 3145, Cell Signaling Technology), EBP50 (1:2000, PA1090, Thermo Fisher Scientific), Rac1 (1:1000, 610651, BD Biosciences), phospho-EGFR (Tyr1068) (1:1000, 2234, Cell Signaling Technology), EGFR (1:1000, 2646, Cell Signaling Technology), mCherry (1:1000, 43590, Cell Signaling Technology), phosphor-PLCγ1 (Tyr783) (1:1000, 2821, Cell Signaling Technology), PLCγ1 (1:1000, 5690, Cell Signaling Technology), phosphor-PKC (pan) (βII Ser660) (1:1000, 9371, Cell Signaling Technology), phospho-AKT1 (S473) (1:1000, 9018, Cell Signaling Technology), AKT1 (1:1000, 2938, Cell Signaling Technology), phospho-p44/42 MAPK (Erk1/2) (Thr202/Tyr204) (1:1000, 9106, Cell Signaling Technology), p44/42 MAPK (Erk1/2) (1:1000, 9102, Cell Signaling Technology), and βA3A1-crystallin (1:1000, ab151722, Abcam), MCT3 (1:1000, AMT013, Alomone Labs), Sodium Potassium ATPase, (1:2000, ab7671, Abcam), Vinculin (1:1000, ab73412, Abcam), β-catenin (1:1000, ab16051, Abcam), phospho-β-catenin (1:1000, 2009, Cell Signaling Technology), β-tubulin (1:1000, 2128S, Cell Signaling Technology), Caveolin-1 (1:1000, 3267, Cell Signaling Technology), Actin (1:1000, A2066, Sigma Aldrich), RIP1 (1:1000, MAB3585, R&D Systems), CLTA (1:1000, 10852-1-AP, Proteintech). Quantification of the band intensity was conducted with Quantity One software (Bio-Rad).

### Statistics and reproducibility

Statistical analysis was performed using GraphPad Prism v.8 software. All graphs show mean ± SD. *P* values were obtained by either two-way ANOVA (multiple comparisons) or a two-tailed unpaired Student’s *t*-test (comparison between two groups). The numbers of biological repeats (*n*) of each experiment are provided in the figure legends. All figures show the data from independent samples or otherwise are indicated in the figure legend as representative data from independent experiments.

### Reporting summary

Further information on research design is available in the Nature Research Reporting Summary linked to this article.

## Supplementary information

Supplementary Information

Description of Supplementary Files

Supplementary Movie 1

Supplementary Movie 2

Supplementary Movie 3

Supplementary Movie 4

Supplementary Movie 5

Supplementary Movie 6

Supplementary Data 1

Reporting Summary

## Data Availability

All data generated or analyzed during this study are included in the article (and its Supplementary Data files). Source data for figures can be found in Supplementary Data 1. Original blots/gel images are shown in Supplementary Fig. 4. All relevant data relating to this manuscript are available upon request. The mass spectrometry proteomics data have been deposited to the ProteomeXchange Consortium via the PRIDE partner repository with the dataset identifier PXD026777.
