# Supplementary material for: βA3/A1-crystallin regulates apical polarity and EGFR endocytosis in retinal pigmented epithelial cells
Source: Commun Biol. 2021 Jul 8;4:850. doi: 10.1038/s42003-021-02386-6 (PMC8266859; doi:10.1038/s42003-021-02386-6)
Supplement: Supplementary file 1 — Supplementary Information [file 42003_2021_2386_MOESM1_ESM.pdf]

Supplementary Information:

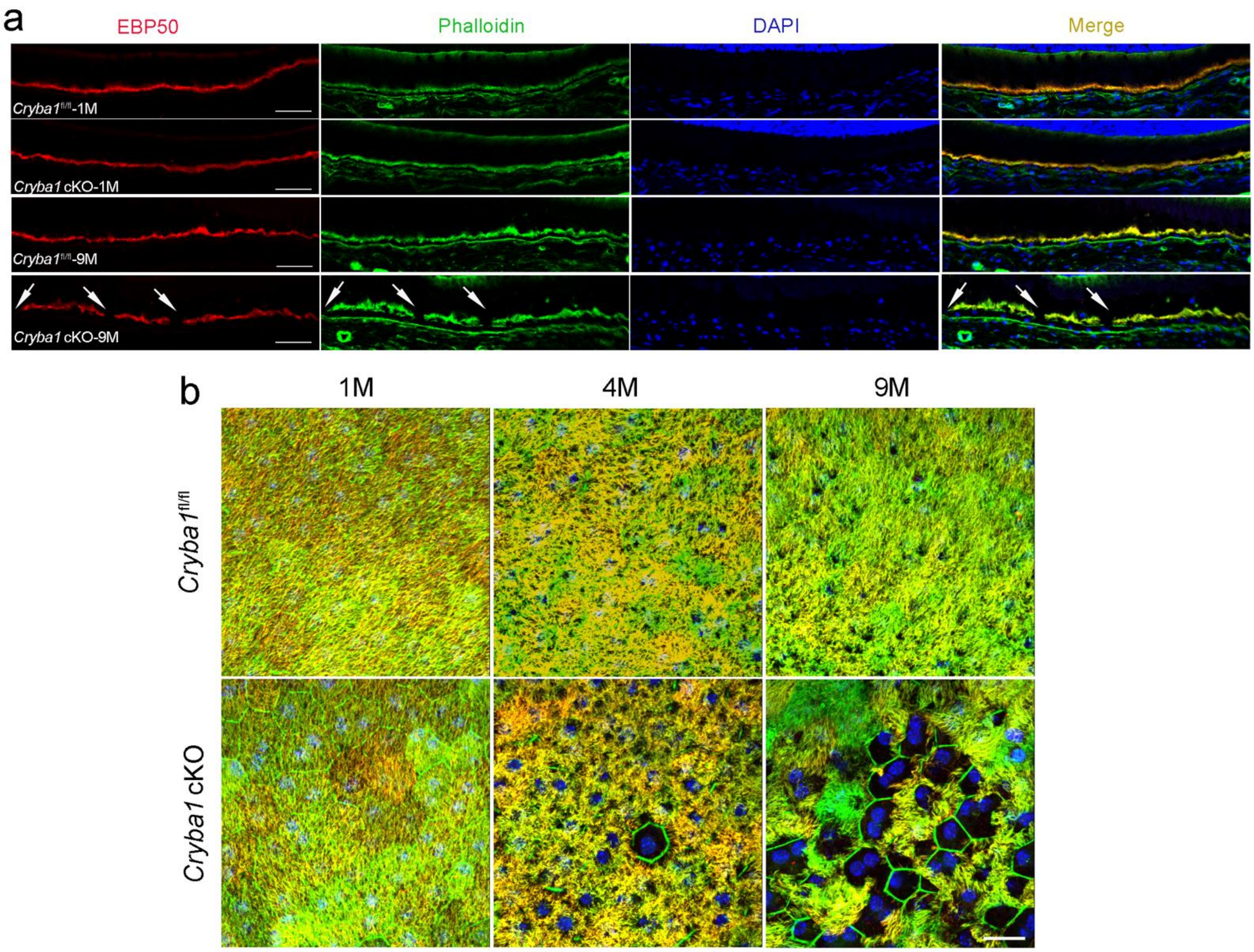

Supplementary Figure 1. *Cryba1* cKO RPE show age-related microvilli defects. (a) Lower magnification images of retina sections immunostained for EBP50 (red) and F-actin (phalloidin, green) show disorganized microvilli in 1-month old cKO RPE cells, and patchy loss of microvilli (arrows) in RPE cells of 9-month old cKO mice compared to age-matched control. DAPI (blue). Scale bar: 50 μm. (b) Lower magnification of the images in Fig 1b are shown in the maximum intensity projection of Z-stack imaging. Images show the merge of EBP50 (red), F-actin (phalloidin, green), and DAPI (blue) in RPE cells from 1-month, 4-month and 9-month old control and cKO mice. The results showed patchy microvilli abnormalities in cKO RPE cells. Scale bar: 20 μm.

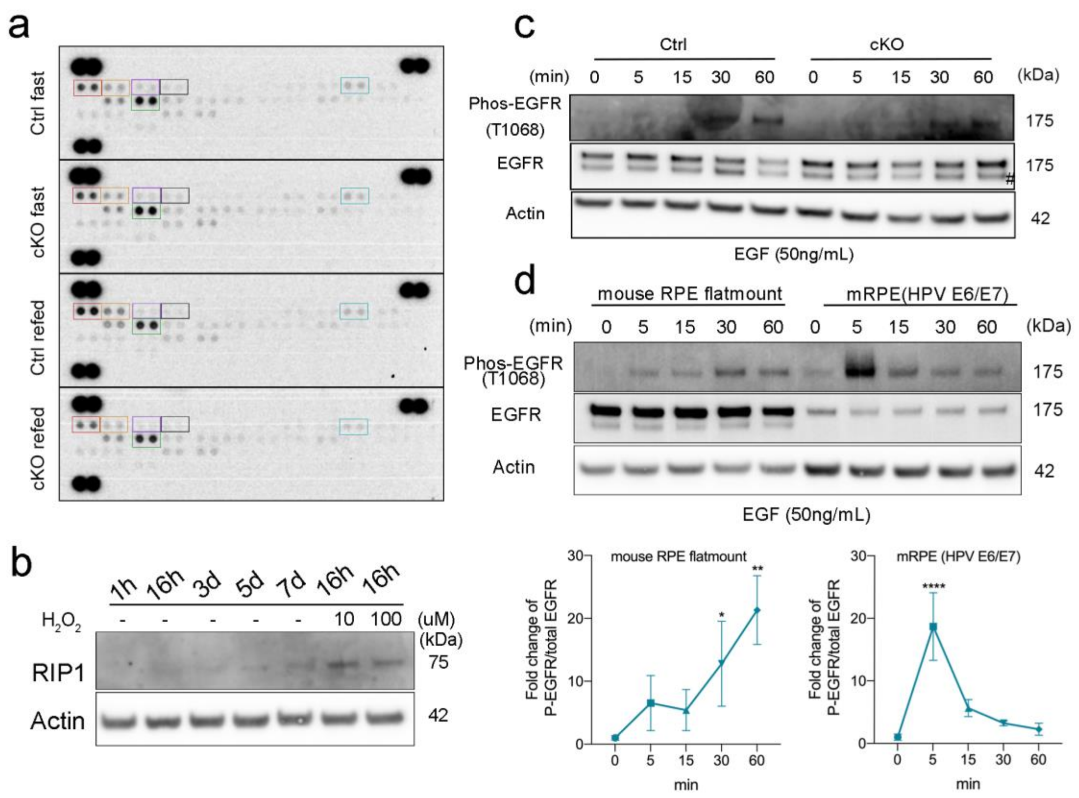

<u>Supplementary Figure 2</u>. Compromised EGFR activation in *Cryba1* cKO RPE. (a) RPE lysates from 2-month old control and cKO mice fasted or fasted and refed were analyzed using a phospho-receptor tyrosine kinase array assay. Red, orange, purple, black, cyan and green boxes indicate phosphorylated EGFR, ErbB2, ErbB3, ErbB4, Axl and PDGFRα in duplicate, respectively. (b) Lysates from RPE flat-mounts cultured *in vitro* for indicated time points were analyzed by western blotting. RIP1 levels were tested in these samples. RPE flat-mounts treated with 10μM and 100μM hydrogen peroxide were used as positive controls for RIP1 detection. (c) Another set of RPE flat-mounts of 2-month old control and cKO mice stimulated with EGF for times indicated were analyzed by western blot. Data showed a similar result as Figure 4c. #: non-specific band. (d) RPE flat-mounts from 3-month old C57BL/6J mice and a mouse RPE cell line (HPV E6/E7) were stimulated with 50ng/mL EGF for indicated times. Western blot analysis showed mRPE (HPV E6/E7) cells exhibited peak EGFR phosphorylation at 5 min after ligand stimulation. However, RPE flat-mounts consistently showed increased EGFR phosphorylation at later time points. Statistical analysis was performed using 1-way ANOVA. **P<0.01, ****P<0.0001. n=3.

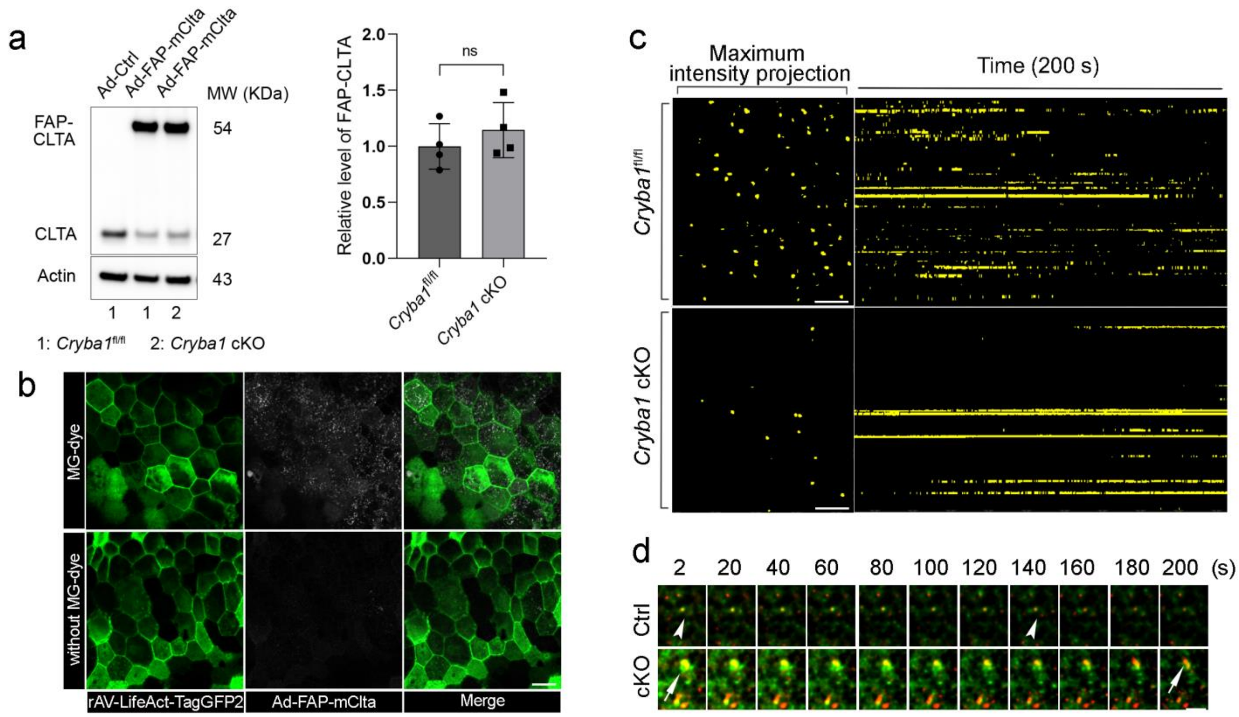

<u>Supplementary Figure 3</u>. Compromised EGFR internalization in *Cryba1* cKO RPE. (a) RPE flatmounts from 2-month old control and cKO mice were infected with Ad-CMV-mClta-FAP and rAV-CMV-LifeAct-TagGFP2. RPE lysates were collected, and the infection efficiency was analyzed by immunoblotting for FAP-CLTA. Western blot data suggested similar infection efficiency for control and cKO RPE flatmounts. Statistical analysis was performed using a two-tailed unpaired Student's t-test. ns=not significant. (b) Confocal images on 2-month old control RPE flatmounts infected with Ad-CMV-mClta-FAP and rAV-CMV-LifeAct-TagGFP2 with or without MG-dye. Scale bar: 20 μm (c) Kymograph of co-localized clathrin with FNBP1. RPE flat-mounts from 2-month old control and cKO mice were infected with Ad-CMV-mClta-FAP, rAV-CMV-LifeAct-TagGFP2, and Ad-CMV-mCherry-mFNBP1 and stimulated with EGF for TIRF imaging. Co-localization of FNBPs and clathrin is indicated by yellow dots. Long lines in the kymograph indicated CCPs which were not released from the cell membrane. Scale bar: 5 μm (d) The track of one CCP (green) co-localized with FNBP1 (red) over time in control and cKO RPE, respectively. Scale bar: 2.5 μm.

Fig 2a

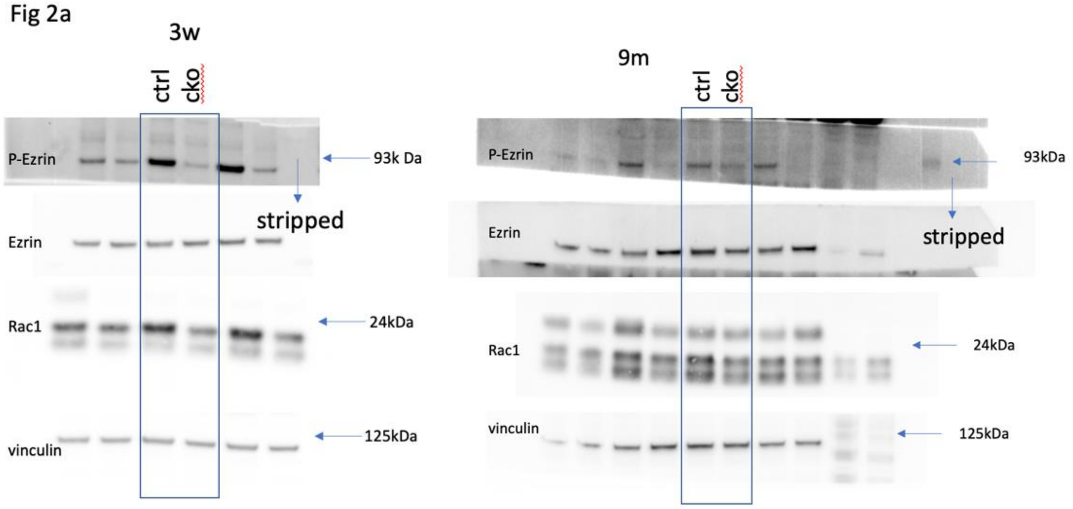

Fig 2b

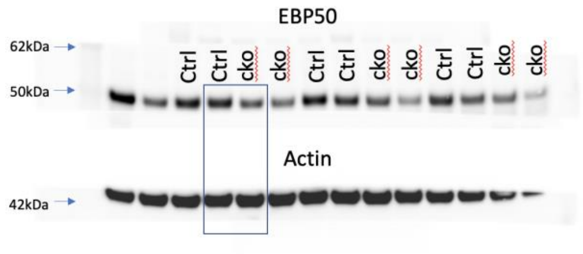

Fig 2d

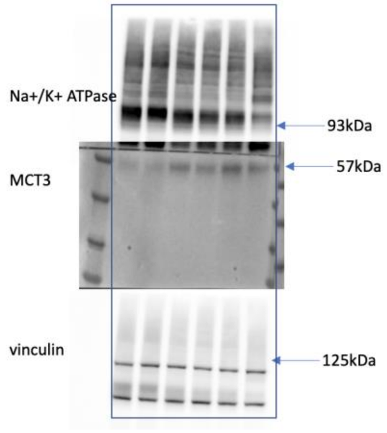

Fig 3

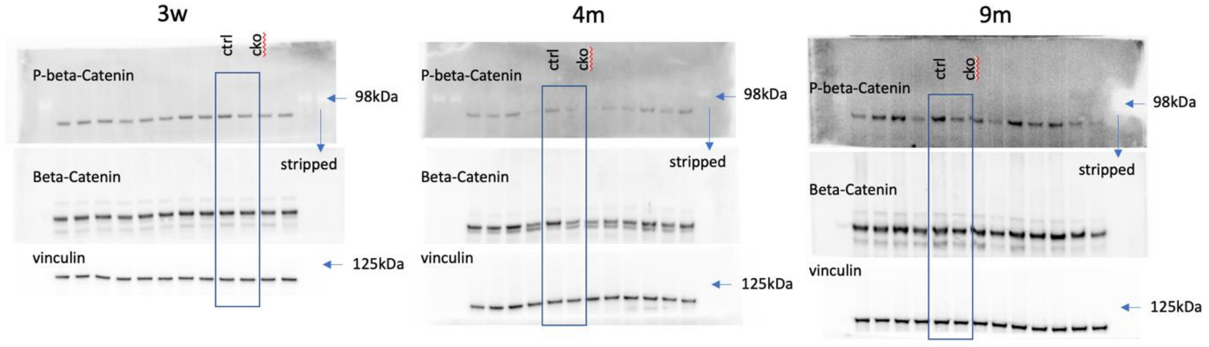

Fig 4c

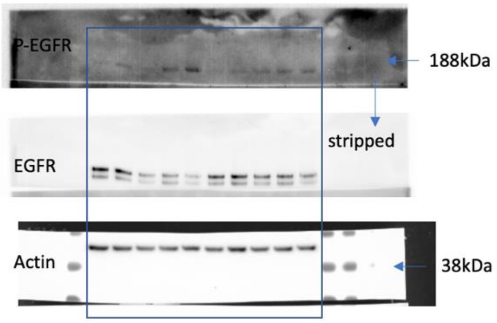

Fig 4d

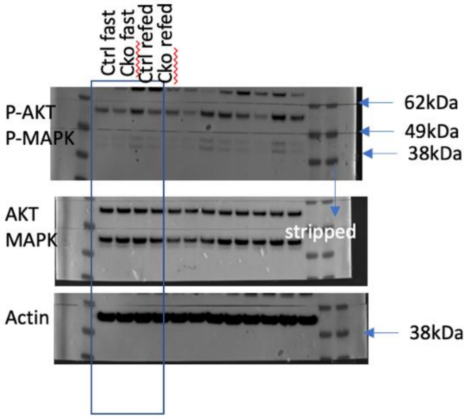

Fig 5h

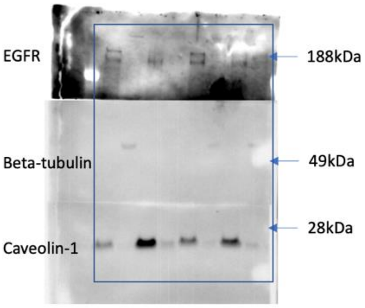

Fig 6a

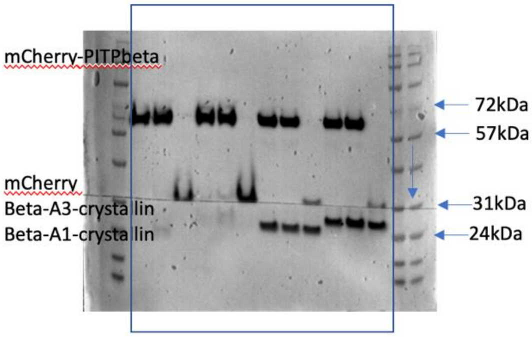

Fig 6e

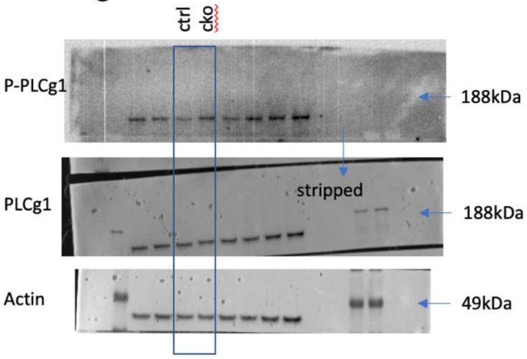

Fig 6f

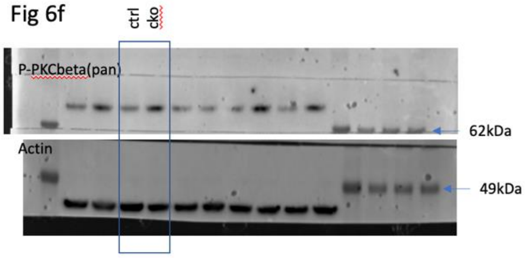

Fig 7a

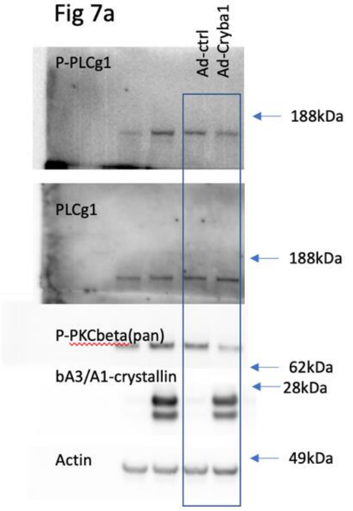

Fig 7b

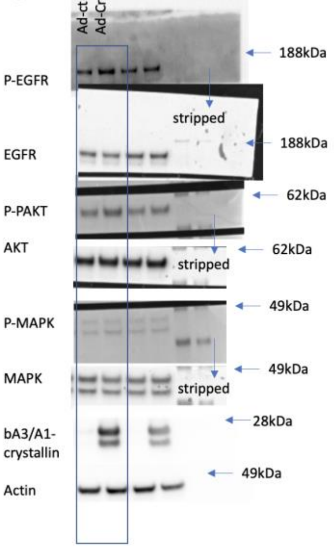

Fig 7e

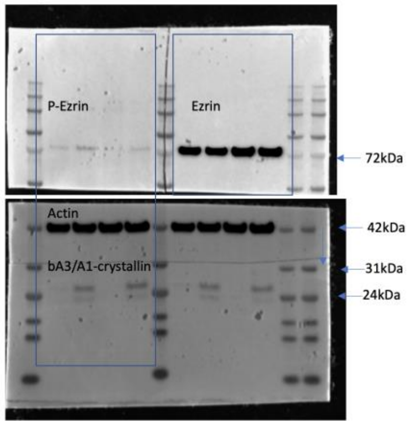

Fig S2b

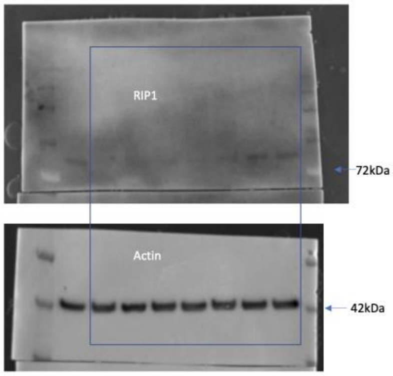

Fig S2c

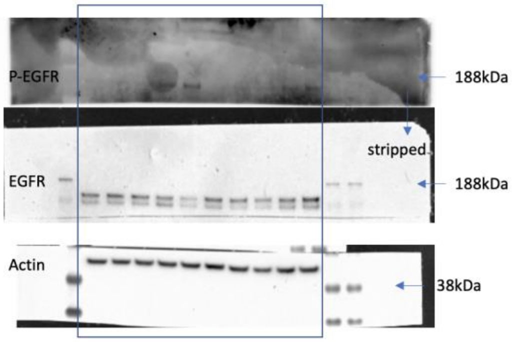

Fig S2d

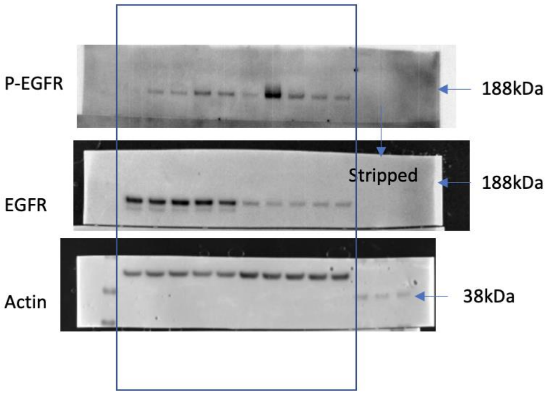

Fig S3a

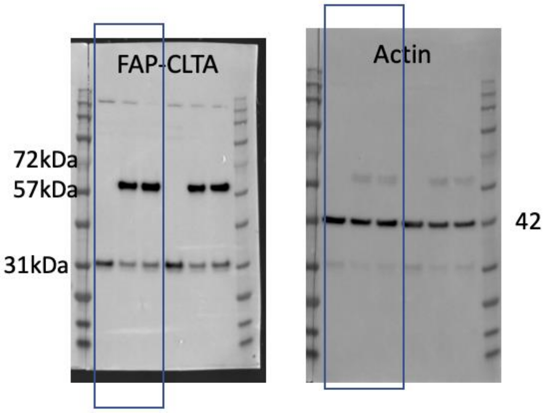

Supplementary Figure 4. This figure shows original blots for all data for each figure panel represented in the manuscript. The bands incorporated in the manuscript are highlighted with blue insets in the raw images.

Sequence	Gene Symbol	Full Name	Modifications	Fold change	Subcellular location
tPSNsSVQPPGLTEEAPGPASPTPPR	SREC2	Scavenger receptor class F member 2	N-Term(TMT6plex); S5(Phospho)	1.55	Membrane
tVsDNsLSSSk	FNBP1	Formin-binding protein 1	N-Term(TMT6plex); S3(Phospho); S6(Phospho); K11(TMT6plex)	1.55	Cytoplasm
sSsLDmNR	REPS1	RalBP1-associated Eps domain-containing protein 1	N-Term(TMT6plex); S3(Phospho); M6(Oxidation)	1.47	Membrane, clathrin-coated pit
sDsQQAVk	SNX17	Sorting nexin-17	N-Term(TMT6plex); S3(Phospho); K8(TMT6plex)	1.42	Cytoplasm. Early endosome. Cytoplasmic vesicle membrane
tSsSETEEk	CD2AP	CD2-associated protein	N-Term(TMT6plex); S3(Phospho); K9(TMT6plex)	1.36	Cytoplasm, cytoskeleton
hsLASTDEk	LRP1	Prolow-density lipoprotein receptor-related protein 1	N-Term(TMT6plex); S2(Phospho); K9(TMT6plex)	1.33	Low-density lipoprotein receptor-related protein 1 85 kDa subunit: Cell membrane
aGGSPAsYHGSTSPR	EPN2	Epsin-2	N-Term(TMT6plex); S7(Phospho)	1.33	Cytoplasm
tVsDNSLSSSk	FNBP1	Formin-binding protein 1	N-Term(TMT6plex); S3(Phospho); K11(TMT6plex)	1.32	Cytoplasm
ysRsSHSGPAEATEk	MRC2	C-type mannose receptor 2	N-Term(TMT6plex); S2(Phospho); S4(Phospho); K15(TMT6plex)	1.28	Membrane; Single-pass type I membrane protein.
sPsPPPDGsPAATPEIR	BIN1	Myc box-dependent-interacting protein 1	N-Term(TMT6plex); S3(Phospho); S9(Phospho)	1.27	Cytoplasm
rLEDsEREETDSEPQAPR	CLN3	Battenin	N-Term(TMT6plex); S5(Phospho)	-1.25	Lysosome membrane
ssHSGPAEATEk	MRC2	C-type mannose receptor 2	N-Term(TMT6plex); S2(Phospho); K12(TMT6plex)	-1.65	Membrane; Single-pass type I membrane protein.
sPSPPPDGsPAATPEIR	BIN1	Myc box-dependent-interacting protein 1	N-Term(TMT6plex); S9(Phospho)	-1.74	Cytoplasm
sGYHDDsDEDLLE	SORT	Sortilin	N-Term(TMT6plex); S7(Phospho)	-1.87	Membrane

Supplementary Table 1. Differentially phosphorylated peptides in *Cryba1* cKO and control RPE cells from 8-month old mice based on a phospho-proteomics assay.
