# Supplementary material for: βA3/A1-crystallin regulates apical polarity and EGFR endocytosis in retinal pigmented epithelial cells
Source: Commun Biol. 2021 Jul 8;4:850. doi: 10.1038/s42003-021-02386-6 (PMC8266859; doi:10.1038/s42003-021-02386-6)
Supplement: Supplementary file 2 — Description of Supplementary Files [file 42003_2021_2386_MOESM2_ESM.pdf]

Description of Additional Supplementary Files

File name: Supplementary Movie 1

Description: Time-lapse imaging of RPE flat-mounts from 2-month old Cryba1fl/fl mice that were infected with Ad-CMV-mClta-FAP and rAV-CMV-LifeAct-TagGFP2 and stimulated with EGF for TIRF microscopy. CCPs were shown as white puncta. Cryba1 fl/fl RPE showed dynamic CCP scission from membrane. Images were taken every 1 second for 15 mins. The video was generated with an interval time of 100ms.

File name: Supplementary Movie 2

Description: Time-lapse imaging of RPE flat-mounts from 2-month old Cryba1 cKO mice that were infected with Ad-CMV-mClta-FAP and rAV-CMV-LifeAct-TagGFP2 and stimulated with EGF for TIRF microscopy. CCPs were shown as white puncta. Some CCPs in cKO RPE stagnated at the apical side of the membrane and did not pinch off from the cell membrane. Images were taken every 1 second for 15 mins. The video was generated with an interval time of 100ms.

File name: Supplementary Movie 3

Description: Time-lapse imaging by HiLo microscopy of RPE flat-mounts from 2-month old Cryba1fl/fl mice infected with Ad-CMV-mClta-FAP and rAV-CMV-LifeAct-TagGFP2 and stimulated with EGF showed the CCV (white puncta) movements beneath the apical surface. Control RPE showed dynamic movement of CCVs. Images were taken every 10ms for 12 seconds. The video was generated in original acquiring time.

File name: Supplementary Movie 4

Description: Time-lapse imaging by HiLo microscopy of RPE flat-mounts from 2-month old Cryba1 cKO mice infected with Ad-CMV-mClta-FAP and rAV-CMV-LifeAct-TagGFP2 and stimulated with EGF showed the CCV (white puncta) movements beneath the apical surface. cKO RPE showed dull CCV movements. Larger CCVs are seen in cKO RPE cells compared to the control. Images were taken every 10ms for 12 seconds. The video was generated in original acquiring time.

File name: Supplementary Movie 5

Description: Time-lapse imaging of RPE flat-mounts from 2-month old Cryba1fl/fl mice that were infected with Ad-CMV-mClta-FAP, rAV-CMV-LifeAct-TagGFP2, and Ad-CMV-mCherry-mFNBP1 and stimulated with EGF for TIRF microscopy. FNBP1 and CLTA were shown in red and green, respectively. Images were taken every 400ms for 200s. The video was generated with an interval time of 100ms.

File name: Supplementary Movie 6

Description: Time-lapse imaging of RPE flat-mounts from 2-month old Cryba1 cKO mice that were infected with Ad-CMV-mClta-FAP, rAV-CMV-LifeAct-TagGFP2, and Ad-CMV-mCherry-mFNBP1 and stimulated with EGF for TIRF microscopy. FNBP1 and CLTA were shown in red and green, respectively. CCPs that were stuck at the apical membrane surface were co-localized with FNBP1 in Cryba1 cKO RPE cells, indicating defective fission in RPE cells of Cryba1 cKO mice. Images were taken every 400ms for 200s. The video was generated with an interval time of 100ms.

File name: Supplementary Data 1

Description: All raw source data.
