# Supplementary material for: βA3/A1-crystallin regulates apical polarity and EGFR endocytosis in retinal pigmented epithelial cells
Source: Commun Biol. 2021 Jul 8;4:850. doi: 10.1038/s42003-021-02386-6 (PMC8266859; doi:10.1038/s42003-021-02386-6)
Supplement: Supplementary file 10 — Reporting Summary [file 42003_2021_2386_MOESM10_ESM.pdf]

nature research

Corresponding author(s): Debasish Sinha

Last updated by author(s): June 18, 2021

Reporting Summary

Nature Research wishes to improve the reproducibility of the work that we publish. This form provides structure for consistency and transparency in reporting. For further information on Nature Research policies, see our Editorial Policies and the Editorial Policy Checklist.

Statistics

For all statistical analyses, confirm that the following items are present in the figure legend, table legend, main text, or Methods section.

n/a	Confirmed	
☐	☒	The exact sample size (*n*) for each experimental group/condition, given as a discrete number and unit of measurement
☐	☒	A statement on whether measurements were taken from distinct samples or whether the same sample was measured repeatedly
☐	☒	The statistical test(s) used AND whether they are one- or two-sided<br>*Only common tests should be described solely by name; describe more complex techniques in the Methods section.*
☐	☒	A description of all covariates tested
☐	☒	A description of any assumptions or corrections, such as tests of normality and adjustment for multiple comparisons
☐	☒	A full description of the statistical parameters including central tendency (e.g. means) or other basic estimates (e.g. regression coefficient) AND variation (e.g. standard deviation) or associated estimates of uncertainty (e.g. confidence intervals)
☐	☒	For null hypothesis testing, the test statistic (e.g. *F*, *t*, *r*) with confidence intervals, effect sizes, degrees of freedom and *P* value noted<br>*Give P values as exact values whenever suitable.*
☒	☐	For Bayesian analysis, information on the choice of priors and Markov chain Monte Carlo settings
☒	☐	For hierarchical and complex designs, identification of the appropriate level for tests and full reporting of outcomes
☒	☐	Estimates of effect sizes (e.g. Cohen's *d*, Pearson's *r*), indicating how they were calculated

Our web collection on statistics for biologists contains articles on many of the points above.

Software and code

Policy information about availability of computer code

Data collection	Zen software (Zeiss), NIS-Elements AR 5.21.01 (Nikon), Azure Biosystems c200 imaging system (Azure Biosystems), Thermo Proteome Discoverer 1.4.1 platform (Thermo Scientific)
Data analysis	Quantity One (Bio-Rad), Excel (Microsoft), GraphPad Prism v8.2 (GraphPad), NIS-Elements AR 5.21.01 (Nikon), Imaris 9.5 (Oxford Instruments)

For manuscripts utilizing custom algorithms or software that are central to the research but not yet described in published literature, software must be made available to editors and reviewers. We strongly encourage code deposition in a community repository (e.g. GitHub). See the Nature Research guidelines for submitting code & software for further information.

Data

Policy information about availability of data

All manuscripts must include a data availability statement. This statement should provide the following information, where applicable:

- Accession codes, unique identifiers, or web links for publicly available datasets
- A list of figures that have associated raw data
- A description of any restrictions on data availability

All data for the manuscript is available in the main text or supplementary files. The mass spectrometry proteomics data have been deposited to the ProteomeXchange Consortium via the PRIDE partner repository with the dataset identifier PXD026777.

Field-specific reporting

Please select the one below that is the best fit for your research. If you are not sure, read the appropriate sections before making your selection.

[x] Life sciences   [ ] Behavioural & social sciences   [ ] Ecological, evolutionary & environmental sciences

For a reference copy of the document with all sections, see nature.com/documents/nr-reporting-summary-flat.pdf

Life sciences study design

All studies must disclose on these points even when the disclosure is negative.

Sample size	Sample sizes was determined based on power calculation, showing a difference of 25% and up to 20% SD, alpha=0.05 and beta=0.2
Data exclusions	No data were excluded, but some samples (<1%) were excluded from the experiments due to technical challenges during tissue collection.
Replication	At least 3 independent experiments were performed to reliably reproduce the results of the experiments.
Randomization	Animals (from respective genotypes) were randomly allocated to each group.
Blinding	To eliminate bias, individuals handling core-facility instruments or performing analysis on specific experiments were blinded to mouse genotype identity as well as the identity of the experimental groups.

Reporting for specific materials, systems and methods

We require information from authors about some types of materials, experimental systems and methods used in many studies. Here, indicate whether each material, system or method listed is relevant to your study. If you are not sure if a list item applies to your research, read the appropriate section before selecting a response.

Materials & experimental systems

n/a	Involved in the study	
[ ]	[x]	Antibodies
[ ]	[x]	Eukaryotic cell lines
[x]	[ ]	Palaeontology and archaeology
[ ]	[x]	Animals and other organisms
[x]	[ ]	Human research participants
[x]	[ ]	Clinical data
[x]	[ ]	Dual use research of concern

Methods

n/a	Involved in the study	
[x]	[ ]	ChIP-seq
[x]	[ ]	Flow cytometry
[x]	[ ]	MRI-based neuroimaging

Antibodies

Antibodies used	phosphor-Ezrin (Thr567), Ezrin, EBP50, Rac1, phospho-EGFR (Tyr1068), EGFR, mCherry, phosphor-PLCγ1 (Tyr783), PLCγ1, phosphor-PKC (pan) (βII Ser660), phosphor-AKT1 (S473), AKT1, phosphor-p44/42 MAPK (Erk1/2) (Thr202/Tyr204) , p44/42 MAPK (Erk1/2), and βA3A1-crystallin, MCT3, Sodium Potassium ATPase, Vinculin, β-catenin, Phospho-β-catenin, β-tubulin, Caveolin-1, Actin, RIP1, CLTA
Validation	The validation information for each antibody are cited in the methods section with catalog numbers provided by the manufacturers.

Eukaryotic cell lines

Policy information about cell lines

Cell line source(s)	Mouse RPE cell line HPV16 E6/E7 was purchase from Applied Biological Materials Inc. (Canada). ARPE19 cell line was purchased from ATCC.
Authentication	ARPE-19 is a spontaneously arising retinal pigment epithelial (RPE) cell line derived in 1986 by Amy Aotaki-Keen from the normal eyes of a 19-year-old male. ARPE-19 expresses the RPE-specific markers CRALBP and RPE-65. The cells are diploid and can be carried for over 30 passages.<br>The Immortalized Mouse Retinal Pigmented Epithelial Cells- HPV E6/E7, derived from healthy C57B1/6 mouse RPE cells, is a cell line that retains many of their in vivo phenotypic characteristics. RPE-specific markers such as RPE65 and cellular retinaldehyde binding protein (CRALBP), as well as epithelial markers ZO1, cytokeratin 8 and 18 are expressed in the Immortalized Mouse Retinal Pigmented Epithelial Cells- HPV E6/E7.
Mycoplasma contamination	No mycoplasma contamination was found during HPV E6/E7 culture.

Commonly misidentified lines (See ICLAC register)	N/A

Animals and other organisms

Policy information about studies involving animals; ARRIVE guidelines recommended for reporting animal research

Laboratory animals	bA3/A1-crystallin conditional knockout (Cryba1 cKO) mice and the control Cryba1fl/fl mice were used in the study.
Wild animals	*Provide details on animals observed in or captured in the field; report species, sex and age where possible. Describe how animals were caught and transported and what happened to captive animals after the study (if killed, explain why and describe method; if released, say where and when) OR state that the study did not involve wild animals.*
Field-collected samples	*For laboratory work with field-collected samples, describe all relevant parameters such as housing, maintenance, temperature, photoperiod and end-of-experiment protocol OR state that the study did not involve samples collected from the field.*
Ethics oversight	All animal studies were conducted in accordance with the Guide for the Care and Use of Animals (National Academy Press) and were approved by the Animal Care and Use Committee of the University of Pittsburgh.

Note that full information on the approval of the study protocol must also be provided in the manuscript.

April 2020
